# Some Synthetic Approaches for Copper-Containing Terpyridine-Functionalized Polysiloxane Complexes

**DOI:** 10.3390/polym18202506

**Published:** 2026-10-13

**Authors:** Sofia S. Kryukova, Elena R. Votchel, Sergey V. Baykov, Vadim P. Boyarskiy, Regina M. Islamova

**Affiliations:** Institute of Chemistry, St. Petersburg State University, 7/9 Universitetskaya Emb., St. Petersburg 199034, Russia; s.kryukova@spbu.ru (S.S.K.); el.wotchel@gmail.com (E.R.V.); s.baykov@spbu.ru (S.V.B.); v.boiarskii@spbu.ru (V.P.B.)

**Keywords:** copper complex, polysiloxane, terpyridine, hydrosilylation reaction, azide–alkyne cycloaddition, redox activity, mechanochemical reaction

## Abstract

Terpyridine-containing polysiloxanes (**tpy-PMHS** and **tpy-PDMS**) were synthesized via hydrosilylation and used as macromolecular ligands for coordination with copper(I) and copper(II) precursors. As a result, a series of novel polymer–metal complexes (**Cu-tpy-PMHS** and **Cu-tpy-PDMS**) with diverse architectures (cross-linked and end-functionalized polymers) and different copper oxidation states were obtained. These polymer–metal complexes were comprehensively characterized by solid-state NMR, UV–vis, X-ray photoelectron spectroscopy and thermogravimetric analysis. The redox activity of the cross-linked **Cu-tpy-PMHS-2** sample was investigated using cyclic voltammetry. **Cu-tpy-PDMS-1** exhibited catalytic activity in mechanochemical azide–alkyne cycloaddition, affording the target product in 80% yield.

## 1. Introduction

Copper is a versatile and widely utilized transition metal whose abundance, diverse coordination chemistry, and broad catalytic potential underpin its extensive applications in chemistry, materials science, and technology [1,2,3]. Particular attention has been devoted to the catalytic applications of copper complexes, which are generally employed in azide–alkyne cycloaddition, oxidation, C–H arylation, and other important reactions [4,5]. Consequently, numerous low molecular weight copper complexes containing various ligands have been developed. Among them, multidentate N-donor ligands, such as bipyridine, phenanthroline, and terpyridine, have attracted significant interest due to their ability to stabilize copper centers in both the +1 and +2 oxidation states [2].

Recent advances in polymer chemistry and materials science have demonstrated that the incorporation of metal-coordinating moieties into polymer architectures represents a promising strategy for the development of polymer–metal complexes (PMCs), combining the functional properties of metal complexes with the advantages of polymeric materials [6,7]. In this context, polymers provide versatile matrices due to their high flexibility, and tunable architectures, enabling their widespread use in engineering, packaging, biomaterials, and robotics [8,9,10,11]. The introduction of metal centers into polymers can impart additional functionalities, including catalytic activity, redox-active properties, sensory ability, and enhanced thermal stability, thereby expanding the potential applications of polymer-based materials [12,13,14].

The well-known copper-containing PMCs have been based on carbon-chain polymer backbones, including polyvinyl alcohol, polystyrene, and polyacrylonitrile, mainly for catalytic applications in oxidative transformations and azide–alkyne cycloaddition reactions [15,16,17]. In contrast, organosilicon polymers, notably polysiloxanes, emerge as promising macromolecular ligands owing to their exceptional flexibility, low glass transition temperature, hydrophobicity, and excellent thermal stability [18,19,20]. Moreover, the accessible functionalization of polysiloxane chains enables the introduction of various metal-coordinating groups, making these polymers suitable for the synthesis of macromolecular ligands.

Despite these advantageous properties, only a limited number of copper-containing PMCs based on polysiloxanes have been reported. For example, copper(II) PMCs based on a hyperbranched polysiloxane have demonstrated catalytic activity in the synthesis of substituted tetrazoles [21]. In another study, aminopropyl-functionalized polysiloxanes were prepared using copper(II) acetate, resulting in the formation of thermochromic PMCs [22].

To the best of our knowledge, terpyridine-functionalized polysiloxanes have not previously been investigated as macromolecular ligands for the development of copper-containing PMCs based on polysiloxanes. The integration of terpyridine coordination sites into a polysiloxane backbone is expected to combine the strong metal-coordination ability of terpyridine [4,23] with unique properties of polysiloxanes, providing a promising strategy for the development of functional copper-containing PMCs. The incorporation of terpyridine units into polysiloxane chains can be achieved through several synthetic approaches. Among them, hydrosilylation seems to be one of the most versatile and widely employed routes [24]. This transformation is commonly used for the functionalization of organosilicon compounds as well as for the cross-linking of polysiloxanes [25]. Another promising strategy is copper-catalyzed azide–alkyne cycloaddition (CuAAC), which proceeds under mild conditions and enables the synthesis of a broad range of functional compounds, including PMCs [26].

Therefore, the development of efficient strategies for the synthesis of copper-containing PMCs based on terpyridine-functionalized polysiloxanes and the evaluation of some of their properties are challenging tasks. The objectives of this study are as follows: (*i*) synthesizing terpyridine-functionalized polysiloxanes with different architectures via hydrosilylation and azide–alkyne cycloaddition reactions; (*ii*) preparing copper-containing PMCs based on terpyridine-functionalized polysiloxanes using copper(I) and copper(II) precursors; (*iii*) characterizing the obtained polymers and PMCs using liquid- and solid-state NMR, FTIR, UV-vis, and X-ray photoelectron spectroscopy; (*iv*) investigating the redox-active properties and thermal stability of the obtained PMCs; and (*v*) evaluating the catalytic activity of the obtained copper-containing PMCs in mechanochemical azide–alkyne cycloaddition reactions.

All experimental data and the corresponding results and discussion are detailed in the following sections.

## 2. Materials and Methods

### 2.1. Materials

Polymethylhydrosiloxane (**PMHS**, *M_n_* ≈ 3200, viscosity 35–45 cSt, 99%, a hydride group Si–H is present in every unit) and hydride-terminated polydimethylsiloxane (**H-PDMS**, *M_n_* ≈ 1000–1100, viscosity 7–10 cSt, 99%) were purchased from ABCR GmbH (Karlsruhe, Germany) and used as received. Karstedt’s catalyst (0.1 M solution of platinum(0)-1,3-divinyl-1,1,3,3-tetramethyldisiloxane complex in xylene) and N,N,N′,N′-tetramethylethane-1,2-diamine (TMEDA, 99%) were also purchased from ABCR GmbH (Karlsruhe, Germany). Phenylacetylene (98%) was bought from Merck KGaA (St. Louis, MO, USA) and distilled under vacuum prior to use.

Solvents CH_2_Cl_2_ (99%), CHCl_3_ (99%), and CH_3_CN (99%) were bought from Vekton (St. Petersburg, Russia) and distilled over P_4_O_10_ (98%, Component-Reaktiv, Moscow, Russia) under an argon atmosphere prior to use. CH_3_OH (99%, Vekton, St. Petersburg, Russia) was distilled over Mg chips with crystalline I_2_ (99%, Chemical Line, St. Petersburg, Russia) under argon. C_6_H_5_CH_3_ (99%) and C_4_H_8_O (THF, 99%) were purchased from NevaReaktiv (St. Petersburg, Russia) and freshly distilled over Na/benzophenone under an argon atmosphere before use. DMF was purchased from NevaReaktiv (St. Petersburg, Russia) and distilled under reduced pressure.

Salts such as anhydrous Na_2_SO_4_ (99%), ethylenediaminetetraacetic acid disodium salt dihydrate (Na_2_EDTA·2H_2_O, 99%), and NaCl (99%) were acquired from Vekton (St. Petersburg, Russia) and used as received. Copper precursors Cu(NO_3_)_2_·3H_2_O and [Cu(CH_3_CN)_4_]BF_4_ were purchased from Chemcraft (Kaliningrad, Russia) and Merck KGaA (St. Louis, MO, USA), respectively. Et_4_NBF_4_ (99%, Merck, St. Louis, MO, USA) was recrystallized from a prepared azeotropic *i*-PrOH/H_2_O mixture (87.5 wt.%) and then from CH_3_CN.

The terpyridine derivative 4′-(4-(prop-2-yn-1-yloxy)phenyl)-2,2′:6′,2″-terpyridine (terpyridine) was synthesized according to the published procedure [27]. Phenyl azide was prepared from aniline following the known procedure [28]. Poly(3-azidopropylmethylsiloxane-*co*-dimethylsiloxane) with 50 mol.% azide-substituted siloxane units (**N_3_-PDMS**) was synthesized via the reaction of the poly(3-chloropropylmethylsiloxane-*co*-dimethylsiloxane) precursors with (*n*-butyl)_4_NN_3_ and NaN_3_, following a slightly modified procedure [29] for both the azidation and purification steps. Gel permeation chromatography (GPC, Shimadzu LC-20 Prominence high-performance liquid chromatography system, Shimadzu, Kyoto, Japan) was used to determine the molecular weight of **N_3_-PDMS**. The number-average molecular weight (*M_n_*) and weight-average molecular weight (*M_w_*) were 670 and 784, respectively, with dispersity (*Đ* = *M_w_*/*M_n_*) of 1.2.

The purity of all compounds was confirmed by nuclear magnetic resonance (NMR) spectroscopy.

### 2.2. Methods

#### 2.2.1. Spectroscopic Characterization

The liquid-state NMR spectra of all compounds were obtained at 25 °C in CDCl_3_ (Chemical Line, St. Petersburg, Russia) using a Bruker Avance III 400 spectrometer (Bruker, Billerica, MA, USA) at resonance frequencies of 400, 125, and 80 MHz, for ^1^H, ^13^C{^1^H}, and ^29^Si{^1^H} nuclei, respectively. Chemical shifts (*δ*) are reported in [ppm] and referenced to the residual signals of non-deuterated solvents: *δ* = 7.26 (^1^H) and 77.2 (^13^C) in CHCl_3_; *δ* = 2.50 ppm (^1^H) and 39.50 ppm (^13^C) in DMSO–*d6*. The following abbreviations are used to designate the multiplicities and intensities of signals: s—singlet, d—doublet, t—triplet, m—multiplet, td—triplet of doublets; H.I.—high intensity. Heteronuclear single quantum coherence (HSQC) and heteronuclear multiple bond correlation (HMBC) spectra were recorded using a Bruker Avance NEO 500 spectrometer (Bruker, Billerica, MA, USA) to establish correlations between ^1^H and ^13^C nuclei.

The solid-state NMR spectra (SSNMR) were recorded on a Bruker Avance III 400 MHz WB (Bruker, Billerica, MA, USA) operating at 80 MHz for ^29^Si{^1^H} (2000 scans) under magic-angle spinning conditions (at 12,500 Hz). The spectra were registered using cross-polarization (with a recycle delay of 2 s and a contact time of 2.0 ms) with ^1^H decoupling. The samples were prepared as powders, and the spectra were referenced to an external standard, pure (CH_3_)_4_Si. The data were processed using MestReNova desktop NMR data processing software (version 6.0.2).

The Fourier-transform infrared (FTIR) spectra were recorded using the attenuated total reflectance technique on a Thermo Scientific Nicolet 8700 FTIR spectrophotometer (Thermo Fisher Scientific, Waltham, MA, USA). The spectra were collected over the range 500–3500 cm^−1^.

The ultraviolet–visible (UV–vis) spectra of PMCs were recorded in distilled CH_3_CN and DMF using a Shimadzu UV-1800 spectrophotometer (Shimadzu, Kyoto, Japan) over the wavelength range of 250–1000 nm at room temperature (r.t., 21 °C).

The copper content of PMCs was measured by energy-dispersive X-ray spectroscopy (EDX) using a Shimadzu EDX-800P spectrometer (Shimadzu, Kyoto, Japan) equipped with a Rh-target X-ray tube (50 W). X-ray photoelectron spectroscopy (XPS) measurements were performed using an Escalab 250Xi photoelectron spectrometer (Thermo Fisher Scientific, Waltham, MA, USA) with AlK_α_ radiation (photon energy = 1486.6 eV). The spectra were acquired in constant pass energy mode using a pass energy of 100 eV for survey scans and 50 eV for element core-level spectra. The XPS measurements were performed with a spot size of 100 μm.

#### 2.2.2. Thermogravimetric Analysis

Thermogravimetric analysis (TGA) of polysiloxanes was carried out on the NETZSCH TG 209 F1 Libra TGA209F1D-0024 analyzer (NETZSCH Group, Selb, Bavaria) in air. The samples were heated from 25 to 1000 °C with a heating rate of 10 °C∙min^−1^.

#### 2.2.3. Electrochemical Measurements

Electrochemical measurements were carried out using cyclic voltammetry (CV) on a SmartStat PS30 (SmartStat LLC, Chernogolovka, Russia) potentiostat/galvanostat at different scan rates (0.05–0.1 V·s^−1^) within the potential range from −2.0 to +0.9 V. The solution of Et_4_NBF_4_ in dried CH_3_CN (0.1 M) was utilized as a supporting electrolyte. A three-electrode electrochemical cell was used. Glassy carbon (GC) electrode (S = 0.07 cm^2^, Shanghai Precision & Scientific Instrument Co. Ltd., Shanghai, China) was used as a working electrode. Prior to the CV measurements, a dispersion of **Cu-tpy-PMHS-2** was drop-cast onto the working electrode and heated at 100 °C. Nonaqueous Ag|AgNO_3_ electrode (MF-2062, Bioanalytical Systems, West Lafayette, IN, USA, internal solution of 0.1 M Et_4_NBF_4_ and 5 mM AgNO_3_ in CH_3_CN; −0.18 V vs. Fc/Fc+) and Pt plate (S = 1.0 cm^2^) were used as a reference and counter electrodes, respectively. The measurements were conducted under a constant stream of argon.

#### 2.2.4. Mechanochemical Experiments

Mechanochemical reactions were carried out using a vibrational ball mill (Retsch Mixer Mill MM 400, Retsch GmbH, Haan, Germany, max. speed 30 Hz) using a stainless-steel jar (1.5 mL) and one stainless-steel ball (5 mm in diameter).

#### 2.2.5. Gel Permeation Chromatography

Molecular weight characteristics (*M_n_*, *M_w_*, and *Ð*), were determined by gel permeation chromatography (GPC) using a Shimadzu LC-20 Prominence high-performance liquid chromatograph system (Shimadzu, Kyoto, Japan) equipped with a refractive index detector and Waters Styragel HT4 analytical column (7.8 mm × 300.0 mm, 10 μm; Waters, Wilmslow, Cheshire, UK). Measurements were performed at 40 °C using THF as the eluent. Polymer solutions (2 mg⋅mL^−1^) were prepared in dry THF and filtered through 0.45 μm polyvinylidene fluoride membrane (Macherey–Nagel GmbH & Co. KG, Duren, Germany) prior to analysis. Molecular weight characteristics were calculated using Shimadzu LabSolutions LC software (version 5.90) based on a calibration curve generated with polystyrene standards (*M_w_* = 2000–62,000).

### 2.3. General Synthetic Procedures

#### 2.3.1. Synthesis of Terpyridine-Containing Polysiloxane Ligands via Hydrosilylation Reaction

To modify the polymer chain with a terpyridine moiety, hydride-containing polysiloxanes were used: polymethylhydrosiloxane (**PMHS**) and hydride-terminated polydimethylsiloxane (**H-PDMS**). Hydrosilylation reaction was carried out in the presence of Karstedt’s catalyst using 4′-(4-(prop-2-yn-1-yloxy)phenyl)-2,2′:6′,2″-terpyridine, which contains an unsaturated C≡C bond in its structure.

**tpy-PDMS**. The synthesis of terpyridine-terminated polysiloxane (**tpy-PDMS**) involved the following steps. A suspension of 4′-(4-(prop-2-yn-1-yloxy)phenyl)-2,2′:6′,2″-terpyridine (44 mg, 0.12 mmol) in dry C_6_H_5_CH_3_ (4 mL) was placed in a Schlenk tube under an argon atmosphere. A solution of Karstedt’s catalyst (70 μL of 0.1 M solution in xylene) was added, and the mixture was stirred for 30 min, during which it turned dark brown. **H-PDMS** (254 mg, 0.12 mmol of hydride groups) in C_6_H_5_CH_3_ (3 mL) was then added dropwise over 30 min under argon. The molar ratio of terpyridine to hydride groups was 1:1. The reaction mixture was stirred at 90 °C for 72 h. The obtained solution was concentrated to 1/3 of its initial volume, and then **tpy-PDMS** was purified through a dissolution–precipitation–decantation process (repeated twice) using CH_2_Cl_2_ as the solvent (1 mL) and cold CH_3_OH as the precipitant (5 mL). Finally, the solvents were removed at 60 °C under reduced pressure (5 mbar), and the target polymer was dried at 5 mbar for 4 h. Yield: 224 mg (75%); dark brown viscous liquid. ^1^H NMR (400 MHz, CDCl_3_, r.t.): δ = 8.73 (d, *J* = 5.0 Hz, 1.7H), 8.71 (s, 1.5H), 8.66 (d, *J* = 8.0 Hz, 2.0H), 7.87 (t, *J* = 8.0 Hz, 3.5H), 7.34 (t, *J* = 5.0 Hz, 1.9H), 7.11 (d, *J* = 8.8 Hz, 0.4H), 7.02 (d, *J* = 8.8 Hz, 1.4H), 6.33 (td, *J_1_* = 18.9 Hz, *J_2_*= 4.7 Hz 0.2H), 6.07 (d, *J* = 18.9 Hz, 0.2H), 5.93 (s, 0.5H), 5.64 (s, 0.5H), 4.73 (s, 0.9H), 4.64 (d, *J* = 4.7 Hz, 0.3H), 0.08 (s, H.I.). ^13^C{^1^H} NMR (125 MHz, CDCl_3_, r.t.): δ = 159.88, 156.54, 155.94, 149.99, 149.23, 147.03, 141.15, 137.00, 132.07, 130.80, 128.61, 126.18, 123.89, 121.52, 118.45, 115.20, 71.55, 1.18. ^29^Si{^1^H} NMR (80 MHz, CDCl_3_, r.t.): δ = −21.90, −20.67. *M_n_* = 7650, *M_w_* = 8980, *Ð* = 1.2.

**tpy-PMHS**. The reaction with **PMHS** was conducted similarly, except that the molar ratio of terpyridine to **PMHS** was 1:4, according to the procedure described in ref. [30]. Under an argon atmosphere, the terpyridine precursor (79 mg, 0.22 mmol) in dry C_6_H_5_CH_3_ (3.5 mL) was placed in a Schlenk tube. A solution of Karstedt’s catalyst (49 μL of 0.1 M solution in xylene) was then added, and the mixture was stirred for 30 min, during which the color turned dark brown. Subsequently, a solution of **PMHS** (52 mg, 0.87 mmol of hydride groups) in anhydrous C_6_H_5_CH_3_ (1.5 mL) was added dropwise over 10 min under argon. Then the reaction mixture was sealed and stirred at 90 °C for 96 h. To confirm completion of the reaction, an aliquot was taken from the reaction mixture for NMR spectroscopy. The synthesized polymer (**tpy-PMHS**) was not subjected to purification to avoid premature self-cross-linking via dehydrocoupling reaction of remaining hydride groups Si–H [30]. After the reaction, the mixture was concentrated on a rotary evaporator to 1/3 of its initial volume at 60 °C and used directly for further studies. Conversion of C≡CH groups is 99% by liquid-state NMR (the ^1^H NMR spectrum presented in Figure S5 of the Supporting Information); viscous brown liquid.

#### 2.3.2. Complexation of tpy-PDMS with Copper(I) and Copper(II) Derivatives

**Cu-tpy-PDMS-1**. A calculated amount of [Cu(CH_3_CN)_4_]BF_4_ (12 mg, 0.038 mmol) in freshly distilled CH_3_CN (1 mL) was added dropwise to a solution of polymer ligand **tpy-PDMS** (35 mg, 0.038 mmol of terpyridine fragments) in dry CHCl_3_ (1 mL) under argon. The reaction mixture immediately turned dark brown and a precipitate formed. The mixture was stirred at r.t. for 12 h. The solid was then isolated by centrifugation (CM-6M, ELMI, St. Petersburg, Russia, 3000 rpm) and washed successively with CH_3_CN (3 × 5 mL) and H_2_O (3 × 5 mL). The supernatant was decanted, and the remaining solid was dried at 120 °C for 2 h. Yield: 41 mg (80%), dark brown solid.

**Cu-tpy-PDMS-2**. The reaction involving Cu(NO_3_)_2_·3H_2_O and **tpy-PDMS** was conducted under similar conditions. A solution of Cu(NO_3_)_2_·3H_2_O (13 mg, 0.052 mmol) in CH_3_CN (1 mL) was added dropwise to **tpy-PDMS** (38 mg, 0.041 mmol) in CHCl_3_ (1 mL), the mixture immediately turned dark green. After the addition, the reaction was stirred at r.t. for 12 h. The solvents were removed (at 60 °C, 5 mbar), and the residue was dried at 120 °C for 2 h. Yield: 40 mg, green solid.

#### 2.3.3. Complexation of tpy-PMHS with Copper(I) and Copper(II) Derivatives

**Cu-tpy-PMHS-1**. Complexation of the polymer ligand **tpy-PMHS** (0.22 mmol of terpyridine fragments) with [Cu(CH_3_CN)_4_]BF_4_ (69 mg, 0.22 mmol) was performed at r.t. under argon by dropwise addition of the copper salt in freshly distilled CH_3_CN (6 mL) to a solution (1.5 mL) of **tpy-PMHS** in C_6_H_5_CH_3_ obtained from the preceding hydrosilylation reaction. This afforded a brown-green precipitate of the copper PMC. After stirring for 12 h at r.t., the precipitate was separated by centrifugation (3000 rpm) and washed with CH_3_CN (3 × 5 mL) and H_2_O (3 × 5 mL). The supernatant was decanted, and the remaining solid was dried at 120 °C for 2 h. Yield: 141 mg, brown solid.

**Cu-tpy-PMHS-2**. In the case of Cu(NO_3_)_2_·3H_2_O, the complexation was carried out by adding copper(II) salt (68 mg, 0.28 mmol) in CH_3_CN (6 mL) to the C_6_H_5_CH_3_ solution (1.5 mL) of the modified **tpy-PMHS** (0.22 mmol of terpyridine fragments) from the preceding hydrosilylation reaction. This led to the immediate precipitation of a green solid. After 12 h, the resulting PMC was purified following the same procedure as described for **Cu-tpy-PMHS-1**. Yield: 138 mg, green solid.

#### 2.3.4. Catalytic Experiments

Phenyl azide (40.2 mg, 0.34 mmol), phenylacetylene (34.4 mg, 0.34 mmol), NaCl as a filler (50 mg), and the macrocatalyst (**Cu-tpy-PDMS-1**, **Cu-tpy-PMHS-1**, **Cu-tpy-PDMS-2**, **Cu-tpy-PMHS-2**, 10 wt.%) were loaded into a 1.5 mL stainless-steel vessel containing a stainless-steel ball (5 mm). The vessel was then sealed under air and placed in a vibrational ball mill for 2 h at a frequency of 30 Hz. After completion of the reaction, the reaction mixture was washed with CDCl_3_ (6 mL) and centrifuged (6000 rpm, 2 min), resulting in the precipitation of the catalyst together with NaCl. The formation of the target product was confirmed by ^1^H NMR spectroscopy (Figures S7–S9). A control experiment without the macrocatalyst was performed under identical reaction conditions. The catalytic reaction was repeated three times for each experiment, and the corresponding average yields are presented in Table S1.

## 3. Results and Discussion

### 3.1. Synthesis of Copper-Containing PMCs

#### 3.1.1. Synthetic Approaches to Terpyridine-Containing Polysiloxanes as Precursors for PMCs

Hydrosilylation and azide–alkyne cycloaddition reactions are widely employed approaches for the functionalization of organosilicon compounds [26,31,32]. Among these, hydrosilylation involves the addition of Si–H bonds across unsaturated groups, preferably C≡C and C=C bonds. The high catalytic activity of hydrosilylation catalysts (e.g., Karstedt’s catalyst and other platinum-group metal catalysts) facilitates the efficient synthesis and modification of organosilicon polymers, including polysiloxanes, under mild conditions [24]. In addition, hydrosilylation is commonly utilized for the preparation of cross-linked silicone materials from commercially available polysiloxanes containing hydride and vinyl functionalities [25]. Beyond these applications, hydrosilylation provides an effective strategy for introducing coordinating moieties into polysiloxane chains, enabling subsequent metal coordination and the synthesis of PMCs.

Nevertheless, this synthetic approach has several limitations. Competing dehydrocoupling reactions can occur during hydrosilylation in the presence of transition metal catalysts, potentially reducing the efficiency and selectivity of the functionalization process [33]. Furthermore, in some cases, limited regioselectivity can complicate the purification of the desired products [34,35]. Despite these challenges, this synthetic strategy has been successfully employed for the preparation of many functionalized polysiloxanes, which serve as precursors for the subsequent formation of PMCs containing iron, cobalt, and other transition metals [6,30,36].

To overcome some of the limitations associated with hydrosilylation, alternative strategies based on click chemistry, particularly CuAAC, have been explored. This pathway offers several advantages, including high regioselectivity, mild reaction conditions, and the absence of side reactions such as dehydrocoupling [26]. However, azide-containing polysiloxanes are not readily available commercially and often require laboratory preparation, which typically proceeds with only moderate yields and may result in variable molecular weights depending on the synthetic approach (*M_n_* ≈ 1000–90,000) [29,37,38]. Nevertheless, due to the high reactivity of azide group for further functionalization, this methodology has been successfully employed for the synthesis of palladium, platinum, rhenium, and other metal complexes [26,30], as well as a wide range of functional polysiloxanes [39,40].

Among the various functional groups that can be introduced into polysiloxanes using the described approaches, terpyridine represents a particularly attractive coordinating moiety for the construction of PMCs. Terpyridine exhibits strong chelating ability toward metal ions, providing stable complexes with reduced susceptibility to metal leaching [4,23,41]. Compared with mono- and bipyridyl ligands, which have been widely employed for the preparation of PMCs [6,42], terpyridine provides a tridentate coordination environment, resulting in stronger metal–ligand interactions. Furthermore, the 4′-position of terpyridine can be readily functionalized, enabling its incorporation into polymer architectures [43].

Polysiloxanes bearing chelating ligands can be prepared by several synthetic approaches, including polycondensation, which has been successfully employed for the synthesis of bipyridine-functionalized polysiloxanes [42]. In the present study, hydrosilylation and CuAAC reactions were selected as complementary strategies for the functionalization of polysiloxanes. Hydrosilylation enables the direct modification of commercially available hydride-functional polysiloxanes [39], whereas CuAAC was selected based on our previous studies demonstrating its efficiency for the incorporation of various functional derivatives into polysiloxane backbones [30,39,44]. To enable both approaches, terpyridine was functionalized with a terminal alkyne group according to a previously reported procedure [27]. The resulting alkyne-functionalized terpyridine could then be incorporated into polysiloxane chains via either CuAAC or hydrosilylation.

The resulting polymer ligands were subsequently coordinated with copper ions to afford the corresponding PMCs. Among these approaches, the CuAAC route appears particularly attractive for the synthesis of copper-containing PMCs, since this reaction employs copper-based catalysts that are conceptually consistent with the target metal center. Therefore, our synthetic investigation was initially focused on the CuAAC strategy (see Section 3.1.2).

#### 3.1.2. CuAAC Synthetic Approach to Terpyridine-Containing Polysiloxanes

**Figure 1:**
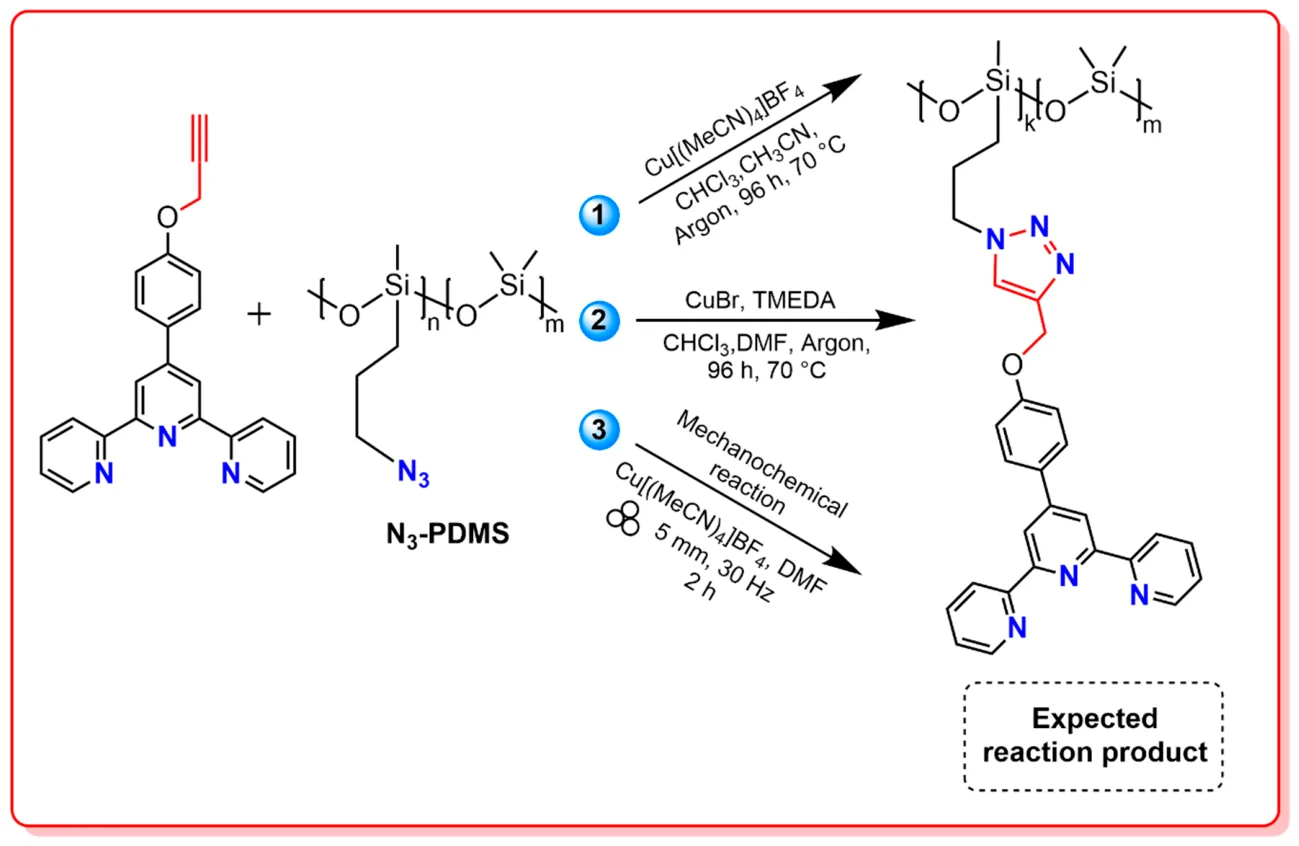
CuAAC approaches for **N_3_-PDMS**.

CuAAC was employed to functionalize azide-containing polysiloxane (**N_3_-PDMS**) with terpyridine coordinating fragments. Several synthetic approaches were investigated (Figure 1). Two copper precursors were evaluated as catalysts for the CuAAC reaction. The first approach employed tetrakis(acetonitrile)copper(I) tetrafluoroborate ([Cu(CH_3_CN)_4_]BF_4_), which has been previously reported as an effective catalyst for CuAAC reaction for modifying polysiloxanes [29]. The second approach utilized copper(I) bromide (CuBr) combined with the coordinating ligand TMEDA, following a reported procedure [45].

In the first experiment (Figure 1, **1**), the reaction conditions were based on a slightly modified version of an earlier reported procedure [30]. **N_3_-PDMS**, the alkyne-containing terpyridine ligand, and copper(I) catalyst ([Cu(CH_3_CN)_4_]BF_4_) were used in a molar ratio of 0.5:1:1, respectively. The reaction was carried out at 70 °C for four days using a CHCl_3_/CH_3_CN solvent mixture under an inert atmosphere. Upon mixing the polymer–ligand (**N_3_-PDMS** and terpyridine) solution with the copper complex, immediate precipitation occurred, accompanied by a color change from dark brown to green. After four days, the precipitate was separated by centrifugation and dried under vacuum. The obtained solid demonstrated solubility in DMSO, and was insoluble in CHCl_3_ and acetone. The ^1^H NMR spectrum (Figure S12) of the precipitate revealed signals attributed to the initial **N_3_-PDMS** (δ = 0.10, 0.59, 1.64 and 3.25, Figure S4) and the CH_2_ group of the terpyridine ligand (δ = 4.76). However, substantial broadening of the aromatic proton signals was observed, which complicated the characterization of the obtained compound. The FTIR spectrum (Figure S13) also did not provide evidence for the formation of the 1,2,3-triazole fragment. Furthermore, only signals corresponding to the initial polymer were observed in the ^1^H NMR spectrum of the soluble fraction (δ = 0.10, 0.59, 1.64 and 3.25).

**Figure 2:**
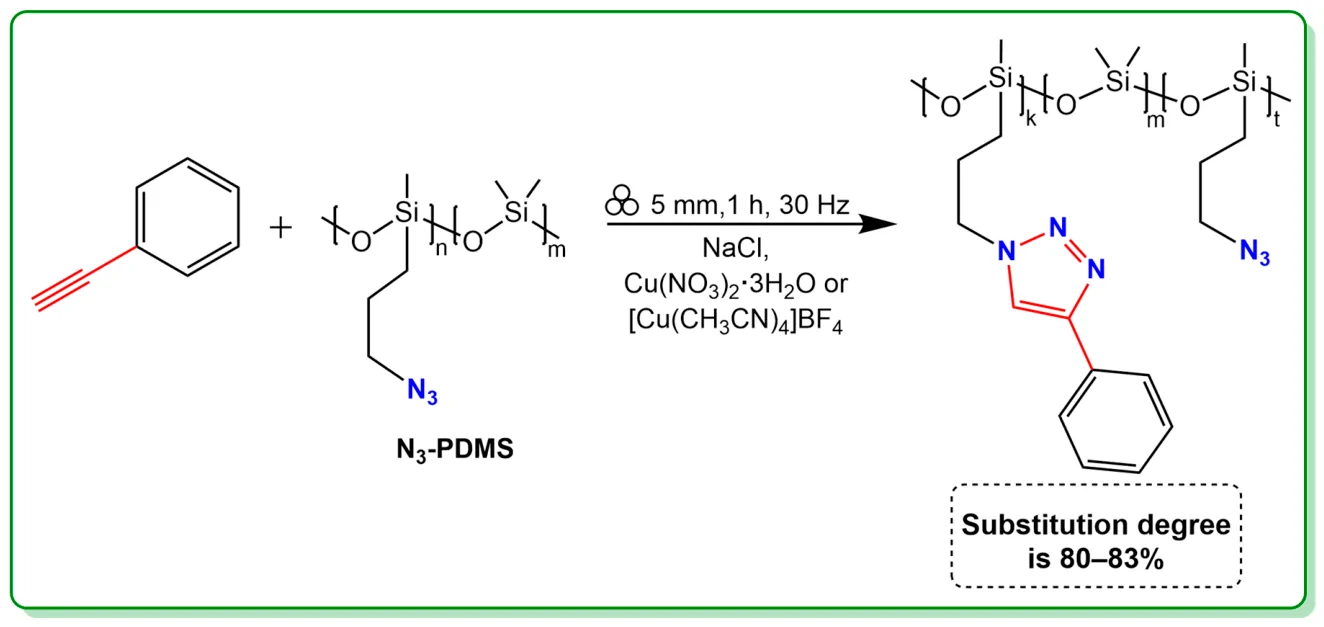
Model reaction of **N_3_-PDMS** and phenylacetylene.

A second synthetic attempt (Figure 1 and Figure 2) involved the use of CuBr as an alternative metal precursor, inspired by its successful application in the CuAAC synthesis of terpyridine-containing polyethylene glycol reported in ref. [45]. In this approach, a DMF/CHCl_3_ solvent mixture was employed, and TMEDA was added as an additional ligand to facilitate copper coordination. The reaction was carried out under similar conditions at 70 °C for four days, during which the formation of the precipitate was also observed. The ^1^H NMR spectrum (Figure S14) of the solid product exhibited features similar to those observed in the first experiment. In particular, substantial broadening of the signals was detected, which hindered the unambiguous confirmation of successful terpyridine incorporation into polysiloxane backbone. Thus, solution-based approaches proved ineffective for the studied polymers.

**Figure 3:**
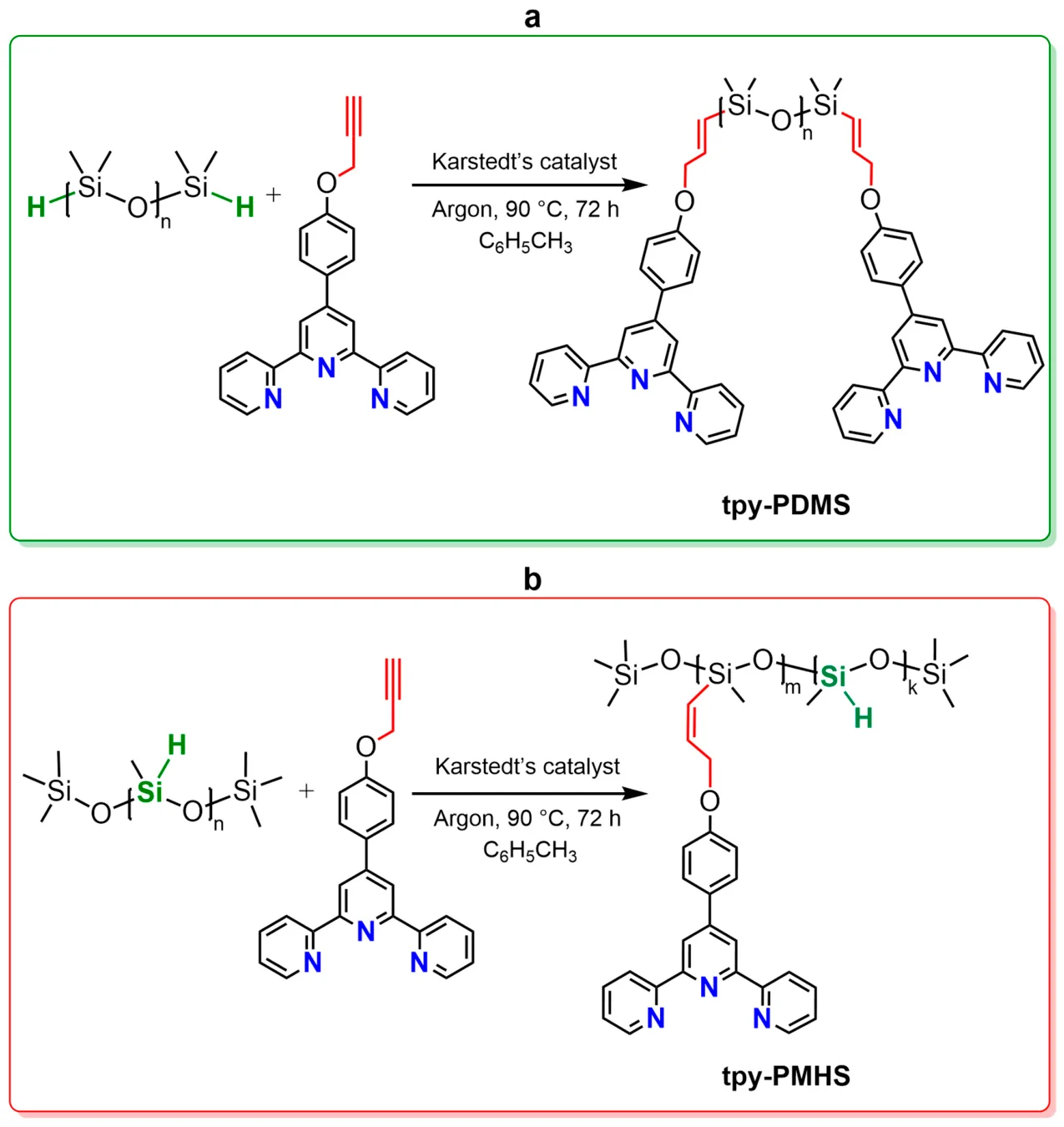
Hydrosilylation reaction of **H-PDMS** (**a**) and **PMHS** (**b**).

Consequently, an alternative synthetic strategy based on mechanochemical preparation was explored (Figure 1 and Figure 3). Mechanochemical synthesis, performed through grinding or milling of reagents, represents a powerful strategy for the rapid and environmentally sustainable synthesis of coordination compounds and polymers [46,47,48]. This approach avoids the use of bulk solvents and overcomes limitations associated with solution-based chemistry, such as solvent coordination [46]. Based on a previous report [40] demonstrating the successful mechanochemical synthesis of copper(I) terpyridine complex, this strategy was investigated as an alternative approach in the present study.

To verify the feasibility of mechanochemical modification of polysiloxanes, a model solvent-free azide–alkyne cycloaddition between the **N_3_-PDMS** and phenylacetylene was first performed (Figure 2). **N_3_-PDMS** (50 mol.% azide groups) and phenylacetylene were milled in equimolar amounts in the presence of either Cu(NO_3_)_2_·3H_2_O or [Cu(CH_3_CN)_4_]BF_4_ at 30 Hz for 1 h. According to ^1^H NMR (Figure S15), the azide–alkyne cycloaddition reached 80–83% conversion under both catalytic systems. These results demonstrate, for the first time, the successful solvent-free mechanochemical functionalization of polysiloxanes via CuAAC reaction.

Following the success of the model reaction, this strategy was extended to the synthesis of terpyridine-functionalized polysiloxanes via mechanochemical approach. The reaction between **N_3_-PDMS**, [Cu(CH_3_CN)_4_]BF_4_, and alkyne-functionalized terpyridine was performed at a 1:1:1 molar ratio. The reaction mixture turned brown during milling, and the product was analyzed by ^1^H NMR spectroscopy (Figure S16). However, this approach also resulted in broadening of the NMR signals, preventing reliable confirmation of terpyridine functionalization. Detailed characterization data are provided in the Supporting Information (Section S3 Synthesis via CuAAC Reaction).

To determine whether the observed changes were related to the interaction between terpyridine and copper(I), rather than to the oxidation of copper(I) to paramagnetic copper(II), a model reaction between the terpyridine ligand and [Cu(CH_3_CN)_4_]BF_4_ was performed. Upon mixing the reagents in DMSO–*d_6_* in an NMR tube under an argon atmosphere, the solution changed color to dark brown within several minutes. However, the ^1^H NMR spectra (Figure S17) recorded at different temperatures (20 and 60 °C) also demonstrated significant broadening of the terpyridine signals.

Therefore, the observed spectral behavior is likely associated with the formation of copper(I)-terpyridine complexes and the averaging effect of dynamic coordination processes involving terpyridine ligands [49]. According to available literature reports, copper(I)- and copper(II)-terpyridine complexes often exhibit challenging spectroscopic behavior [49,50]. For example, complete NMR characterization of a copper(I) complex reported in ref. [49] was achieved only at low temperatures of approximately −100 °C. In the present study, analogous characterization was further complicated by the limited solubility of the obtained substances.

Overall, the performed experiments demonstrated that copper-mediated coordination of terpyridine ligands readily occurs, as evidenced by the characteristic color changes and formation of copper-containing precipitates (the EDX analysis revealed a copper content of 0.4%). However, the successful covalent incorporation of terpyridine units into the polysiloxane structure or the formation of well-defined terpyridine-containing polymers could not be conclusively confirmed. Despite the widespread use of CuAAC reaction for polysiloxane modification [29,37,38], this approach was found to be inappropriate for the present study. Consequently, hydrosilylation was explored as an alternative synthetic route.

#### 3.1.3. Synthesis of Terpyridine-Containing Polysiloxanes via Hydrosilylation

As discussed in the previous section (see Section 3.1.2), hydrosilylation is one of the key strategies for the synthesis and functionalization of polysiloxanes. Polymethylhydrosiloxane (**PMHS**) and hydride-terminated polydimethylsiloxane (**H-PDMS**) with different contents of Si–H group were employed in the present study for the incorporation of terpyridine fragments (Figure 3).

Hydrosilylation was conducted at 90 °C for 72 h in the presence of Karstedt’s catalyst and alkyne-functionalized terpyridine. In the case of **H-PDMS**, the resulting terpyridine-terminated polydimethylsiloxane (**tpy-PDMS**) was purified by reprecipitation from CH_3_OH and characterized by NMR spectroscopy. The successful functionalization of **H-PDMS** was initially confirmed by liquid-state ^1^H NMR spectroscopy. The ^1^H NMR spectrum of **tpy-PDMS** showed characteristic signals corresponding to the terpyridine moiety (δ = 8.73, 8.71, 8.66, 7.87, 7.34, and 7.02 ppm) as well as resonances associated with the formed vinyl groups (δ = 6.33–5.64 ppm, Figure S1). Two sets of vinyl signals were observed, indicating the formation of both *trans*- and *α*-hydrosilylation products [51]. A triplet of doublets at δ = 6.33 ppm and a doublet at δ = 6.07 ppm were tentatively assigned to the vinyl protons of the *trans*-isomer. The large spin–spin coupling constant (*J* = 18.9 Hz) is consistent with the formation of a *trans*-product. Two additional singlets at δ = 5.93 and 5.64 ppm were attributed to the *α*-isomer. Furthermore, signals at δ = 4.73 ppm and δ = 4.63 ppm (doublet) were assigned to the –O–CH_2_– groups associated with the *α*- and *trans*-hydrosilylation products, respectively. The ratio of the *α*- and *trans*-products was determined to be 75:25 based on the relative integration of the characteristic vinyl proton signals in the ^1^H NMR spectrum.

The structures of two hydrosilylation isomers were further verified by two-dimensional HSQC and HMBC NMR spectroscopy (Figure S3). In ^1^H–^13^C HSQC spectrum, the vinyl proton signals of the *α*-isomer at δ = 5.93 and 5.64 ppm showed cross-peaks with a single carbon resonance at δ = 126.20 ppm. In contrast, the vinyl proton signals of the *trans*-isomer at δ = 6.07 and 6.33 ppm correlated with two distinct carbon resonances at δ = 132.07 and 141.15 ppm (Figure S2). The –O–CH_2_– groups associated with both products were also confirmed by correlations with carbon resonances at δ = 71.55 and 70.51 ppm. The HMBC spectrum further supported the presence of both hydrosilylation products by revealing the expected two-bond (*^2^J_CH_*) and three-bond (*^3^J_CH_*) correlations (Figure S3). In the ^29^Si{^1^H} NMR spectrum of **tpy-PDMS**, resonances at δ = −21.90 and −20.67 ppm were assigned to the –[OSi(CH_3_)_2_]– units (Figure S2). FTIR spectroscopy provided further evidence for the reaction by the disappearance of the absorption band at 2118 cm^−1^, corresponding to the C≡C stretching vibration (Figure S6). The number- and weight-average molecular weight of **tpy-PDMS** was determined by GPC analysis (Figure S7) as *M_n_* = 7650 and *M_w_* = 8980 (dispersity *Ð* = 1.2). The noticeable increase in the molecular weight of **tpy-PDMS** compared with the initial **H-PDMS** is likely associated with the extension of polymer chains through a side dehydrocoupling reaction [30].

The reaction with **PMHS** was carried out under similar conditions. Since the molar ratio of terpyridine to **PMHS** was 1:4, the resulting **tpy-PMHS** retained unreacted Si–H groups. These residual hydride functionalities may undergo dehydrocoupling reactions in the presence of Karstedt’s catalyst, leading to cross-linking of the polymer network through the formation of new Si–Si or Si–O–Si bonds with the evolution of hydrogen gas (dehydrocoupling reaction of **tpy-PMHS** is discussed in detail in Section 3.1.4) [33]. The reaction progress was monitored by ^1^H NMR spectroscopy through the analysis of aliquots of the reaction mixture. The disappearance of the signal at δ = 2.56 ppm, assigned to the terminal alkyne proton (C≡CH), confirmed the consumption of the starting alkyne (Figure S5).

As a result, two polymer ligands, **tpy-PDMS** and **tpy-PMHS**, with different architectures were successfully synthesized. The incorporation of the terpyridine moiety into the polysiloxane backbone enabled subsequent metal coordination, leading to the formation of the target PMCs, as discussed in Section 3.1.4.

#### 3.1.4. Preparation and Characterization of Copper-Containing PMCs

**Figure 4:**
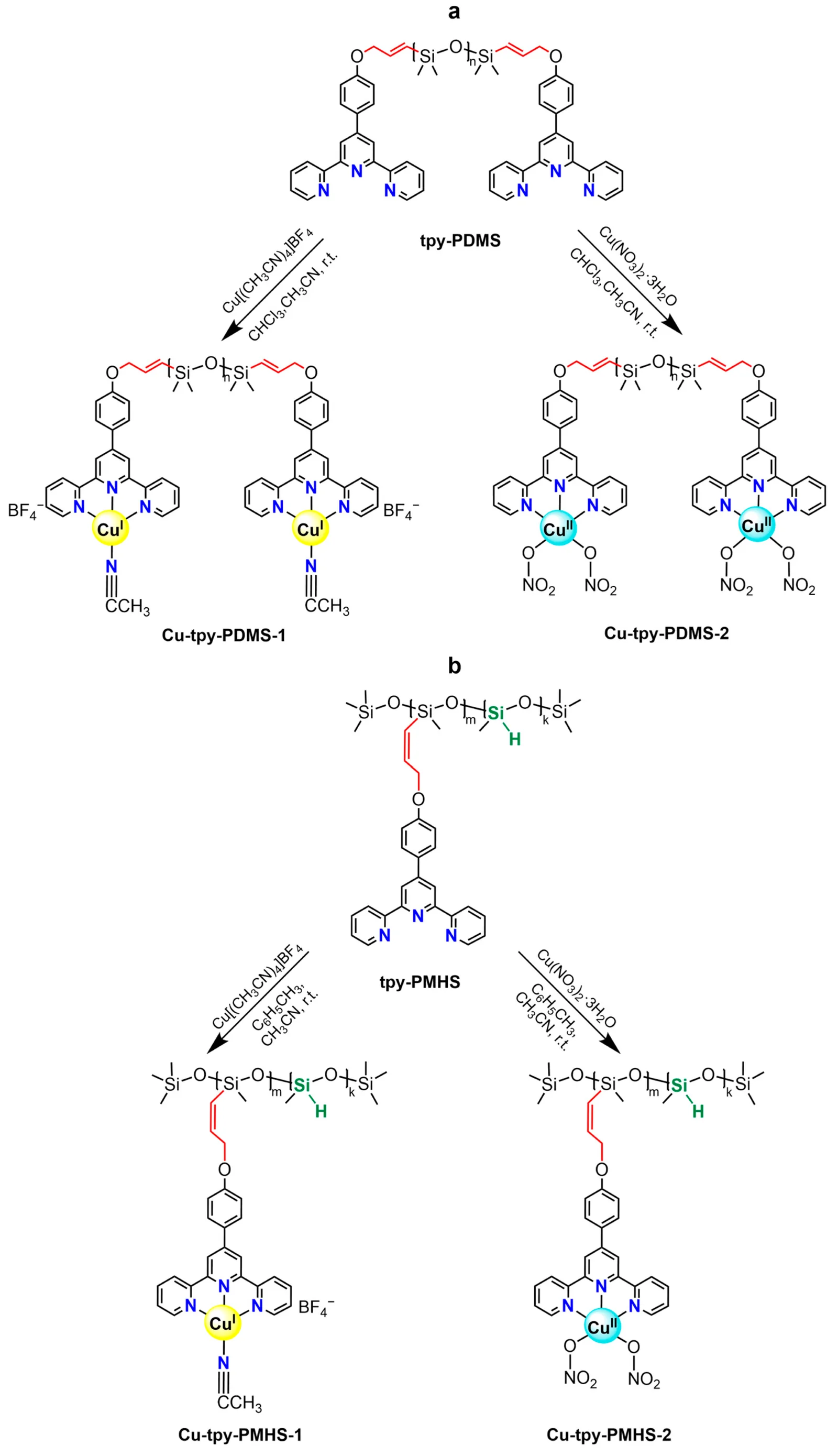
The proposed structures of the resulting PMCs derived from **tpy-PDMS** (**a**) and **tpy-PMHS** (**b**).

Copper-containing PMCs were obtained by coordination of two successfully modified polysiloxanes (**tpy-PMHS** and **tpy-PDMS**) with [Cu(CH_3_CN)_4_]BF_4_ and Cu(NO_3_)_2_·3H_2_O, as precursors of copper(I) and copper(II), respectively. The proposed structures of the resulting PMCs are presented in Figure 4.

Reactions were carried out at r.t. by adding a CH_3_CN solution of the metal precursors to the functionalized polysiloxane ligands. To minimize oxidation of copper(I), reactions involving [Cu(CH_3_CN)_4_]BF_4_ were conducted under an argon atmosphere. In most cases, rapid precipitation of the resulting complexes was observed. The only exception was **Cu-tpy-PDMS-2**, synthesized from **tpy-PDMS** and Cu(NO_3_)_2_·3H_2_O, which remained soluble under the reaction conditions. The precipitated PMCs (**Cu-tpy-PDMS-1**, **Cu-tpy-PMHS-1**, and **Cu-tpy-PMHS-2**) were isolated by centrifugation, washed several times with CH_3_CN and H_2_O to remove unreacted salts, and dried at 120 °C for 2 h. After drying, all samples were obtained as green (**Cu-tpy-PDMS-2**, **Cu-tpy-PMHS-2**) or brown solids (**Cu-tpy-PDMS-1**, **Cu-tpy-PMHS-1**).

**Figure 5:**
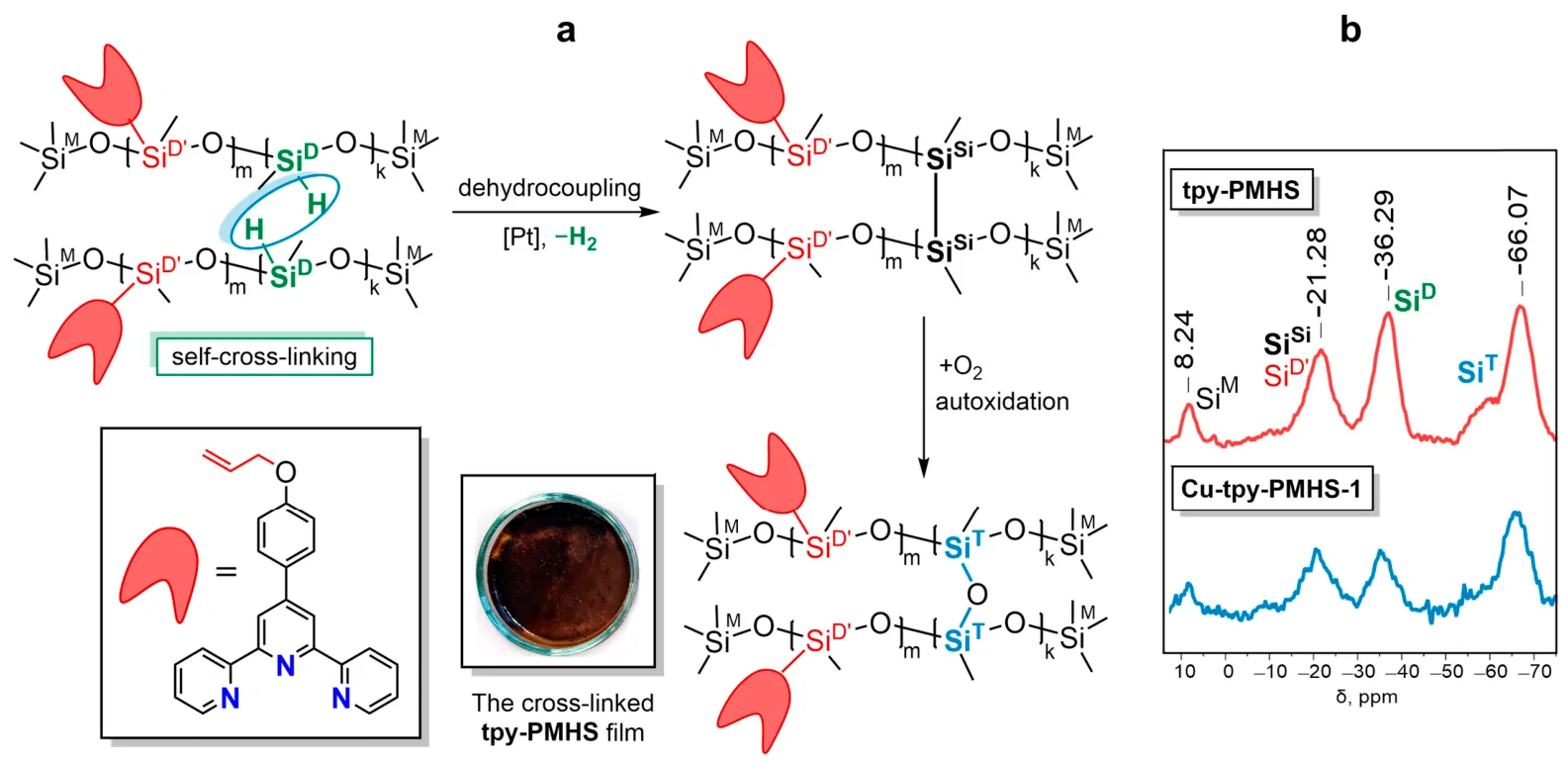
Self-cross-linking reaction of **tpy-PMHS**, along with a corresponding photograph of the **tpy-PMHS** film (**a**); solid-state ^29^Si{^1^H} NMR spectra of **tpy-PMHS** and **Cu-tpy-PMHS-1** (**b**).

For the modified **tpy-PMHS** samples (**Cu-tpy-PMHS-1** and **Cu-tpy-PMHS-2**), heating at 120 °C induced dehydrocoupling of the residual Si–H groups, resulting in a cross-linked material (Figure 5). The formation of Si–Si and Si–O–Si cross-links through dehydrocoupling and subsequent oxidation processes was confirmed by solid-state ^29^Si{^1^H} NMR spectroscopy, which revealed a characteristic signal at δ = −66.07 ppm in both the polymer ligand (**tpy-PMHS**) and the corresponding PMC.

Copper incorporation into the polymer matrices was confirmed by complementary techniques, including spectroscopic methods (EDX, XPS, FTIR, UV–Vis) and electrochemical measurements.

**Figure 6:**
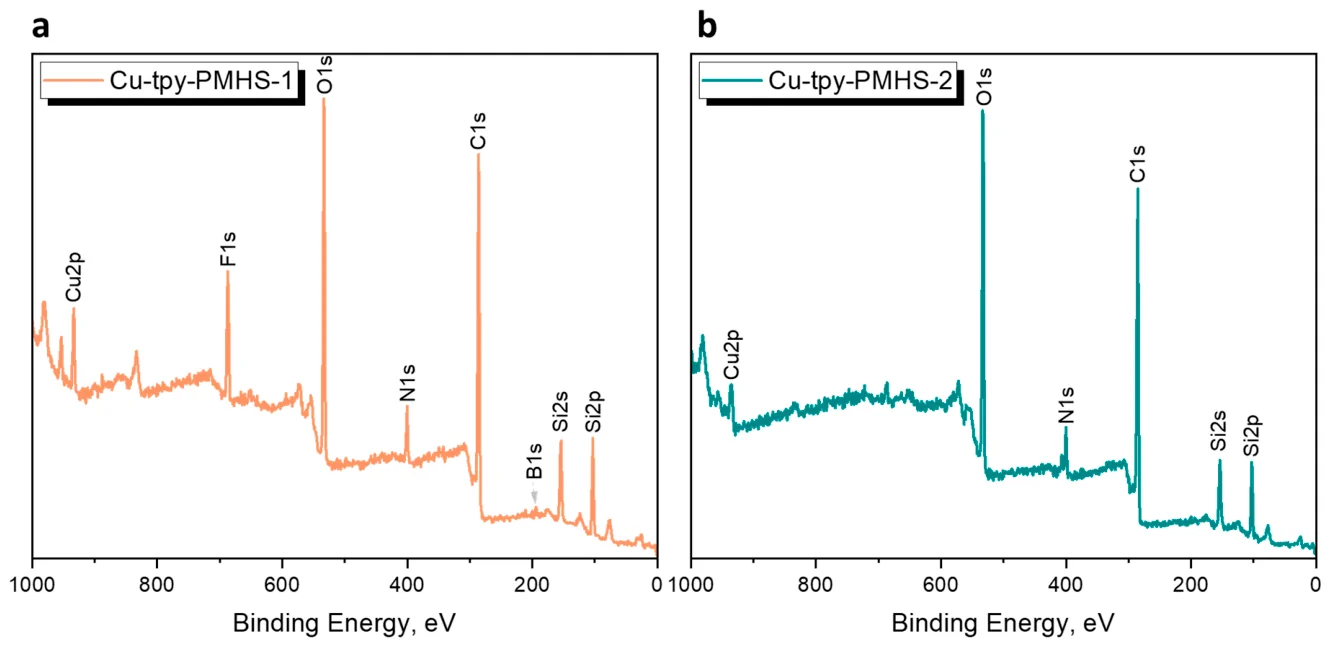
XPS survey spectra of **Cu-tpy-PMHS-1** (**a**) and **Cu-tpy-PMHS-2** (**b**).

The presence of copper in the synthesized and purified PMCs was initially confirmed by elemental analysis using the EDX technique. According to the EDX measurements, **Cu-tpy-PMHS-1** and **Cu-tpy-PMHS-2** contained 8.1 and 5.3 wt.% of copper, respectively. Similarly, **Cu-tpy-PDMS-1** and **Cu-tpy-PDMS-2** exhibited copper contents of 10.5 and 7.0 wt.%. The experimental copper contents were consistent with the theoretical values calculated for the synthesized PMCs, which were 9.6 wt.% and 9.9 wt.% for **Cu-tpy-PMHS-1** and **Cu-tpy-PMHS-2**, respectively, and 6.5 wt.% and 6.7 wt.% for **Cu-tpy-PDMS-1** and **Cu-tpy-PDMS-2**. XPS analysis was further employed to investigate the elemental composition and chemical environment of the synthesized PMCs. The survey XPS spectrum of **Cu-tpy-PMHS-1** (Figure 6) revealed characteristic signals corresponding to C 1s (284.8 eV), O 1s (532.4 eV), N 1s (399.5 eV), Si 2s (154.4 eV), and Si 2p (102.5 eV), which can be assigned to the polysiloxane backbone and terpyridine fragments. A similar elemental composition was observed for **Cu-tpy-PMHS-2**. However, unlike **Cu-tpy-PMHS-2**, the spectrum of **Cu-tpy-PMHS-1** additionally exhibited F 1s (685.5 eV) and B 1s (193.2 eV) signals, originating from the [Cu(CH_3_CN)_4_]BF_4_ complex.

**Figure 7:**
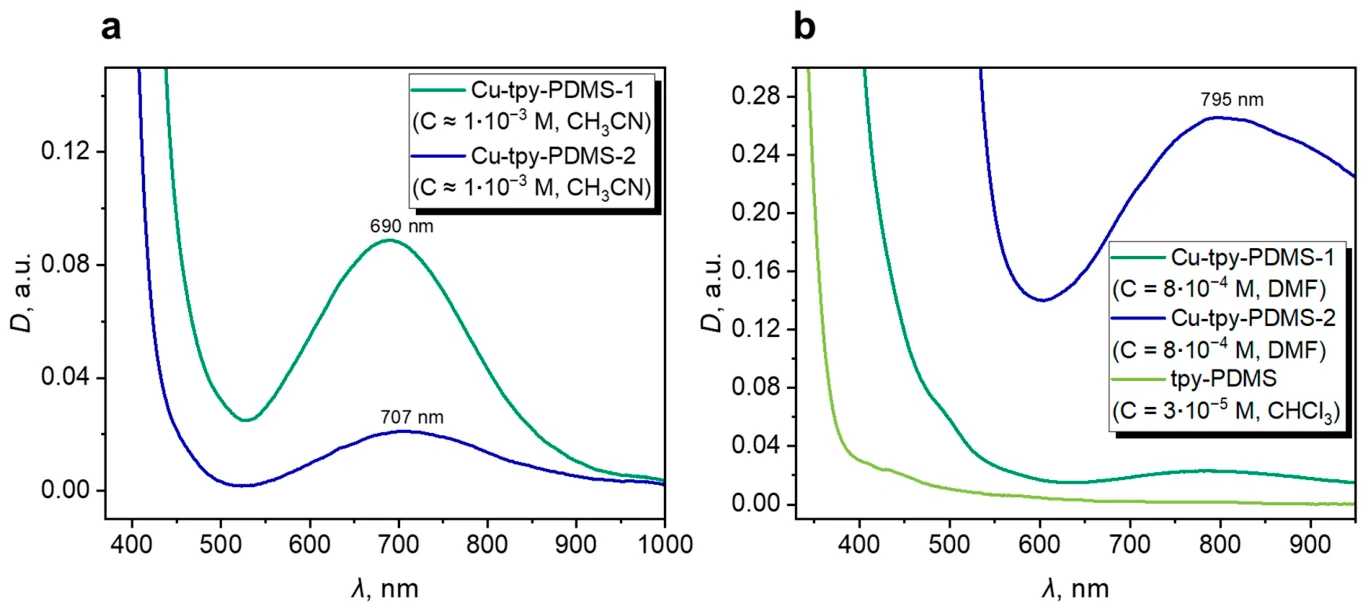
UV–vis spectra of **Cu-tpy-PDMS-1** and **Cu-tpy-PDMS-2** (CH_3_CN and DMF) and **tpy-PDMS** (CHCl_3_) solutions at different concentrations (**a**,**b**).

UV–vis spectra of the soluble **Cu-tpy-PDMS-1** and **Cu-tpy-PDMS-2** samples were recorded in two different solvents, CH_3_CN and DMF. Despite the limited solubility of both PMCs in CH_3_CN, well-defined absorption bands were observed in the 690–707 nm region (Figure 7). For **Cu-tpy-PDMS-2**, synthesized from Cu(NO_3_)_2_·3H_2_O, the band at 707 nm is assigned to a d–d transition, in agreement with reported low molecular weight copper(II) terpyridine complexes [52]. In the case of **Cu-tpy-PDMS-1**, prepared using [Cu(CH_3_CN)_4_]BF_4_, an absorption band centered at 690 nm was observed, which can be tentatively attributed to a metal-to-ligand charge transfer (MLCT) transition. The possibility of partial oxidation of copper(I) during complex isolation or exposure to air cannot be excluded and may also contribute to this absorption band. In DMF, both PMCs dissolved completely, enabling more reliable spectral characterization. Notably, **Cu-tpy-PDMS-2** exhibited a strong broad absorption band centered at approximately 795 nm, characteristic of a Cu(II) d–d transition, whereas this band was absent in the spectrum of the parent **tpy-PDMS** ligand.

The FTIR spectra of the polymer ligands and the corresponding PMCs were found to be almost identical (Figure S6). This is consistent with the limited evidence of complex formation provided by FTIR spectroscopy in previously described low molecular weight copper(II) complexes [50].

The **Cu-tpy-PMHS-2** sample was selected among the synthesized copper-containing PMCs for electrochemical studies because residual Si–H groups allowed the formation of a cross-linked polymer film on the electrode surface. Furthermore, the use of the Cu(II) precursor provided a well-defined initial oxidation state, facilitating interpretation of the redox processes. Therefore, **Cu-tpy-PMHS-2** was selected as a representative copper(II)-containing PMC for electrochemical characterization.

**Figure 8:**
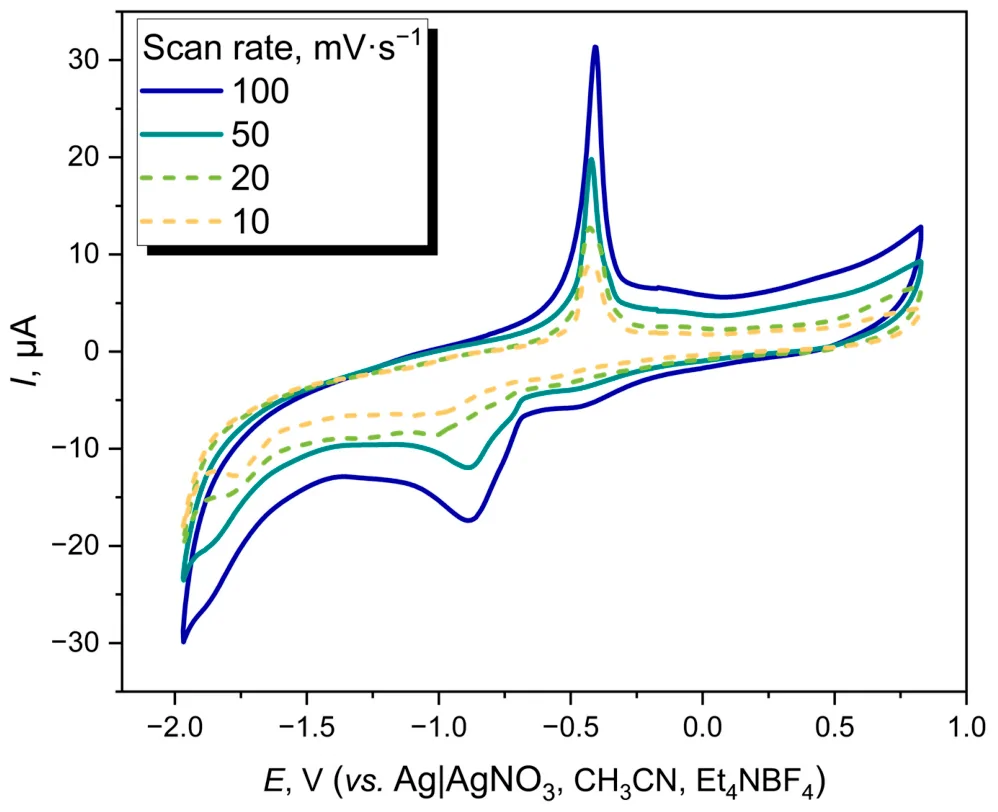
Cyclic voltammograms of **Cu-tpy-PMHS-2.**

CV measurements were carried out in a three-electrode system under an argon atmosphere using a 0.1 M solution of Et_4_NBF_4_ in CH_3_CN as a supporting electrolyte. A dispersion of **Cu-tpy-PMHS-2** was drop-casted onto a GC electrode and heated at 100 °C. The resulting voltammogram (Figure 8) revealed a cathodic peak at approximately −0.88 V and a corresponding anodic peak at −0.42 V, which can be assigned to the Cu(II)/Cu(I) redox couple.

**Figure 9:**
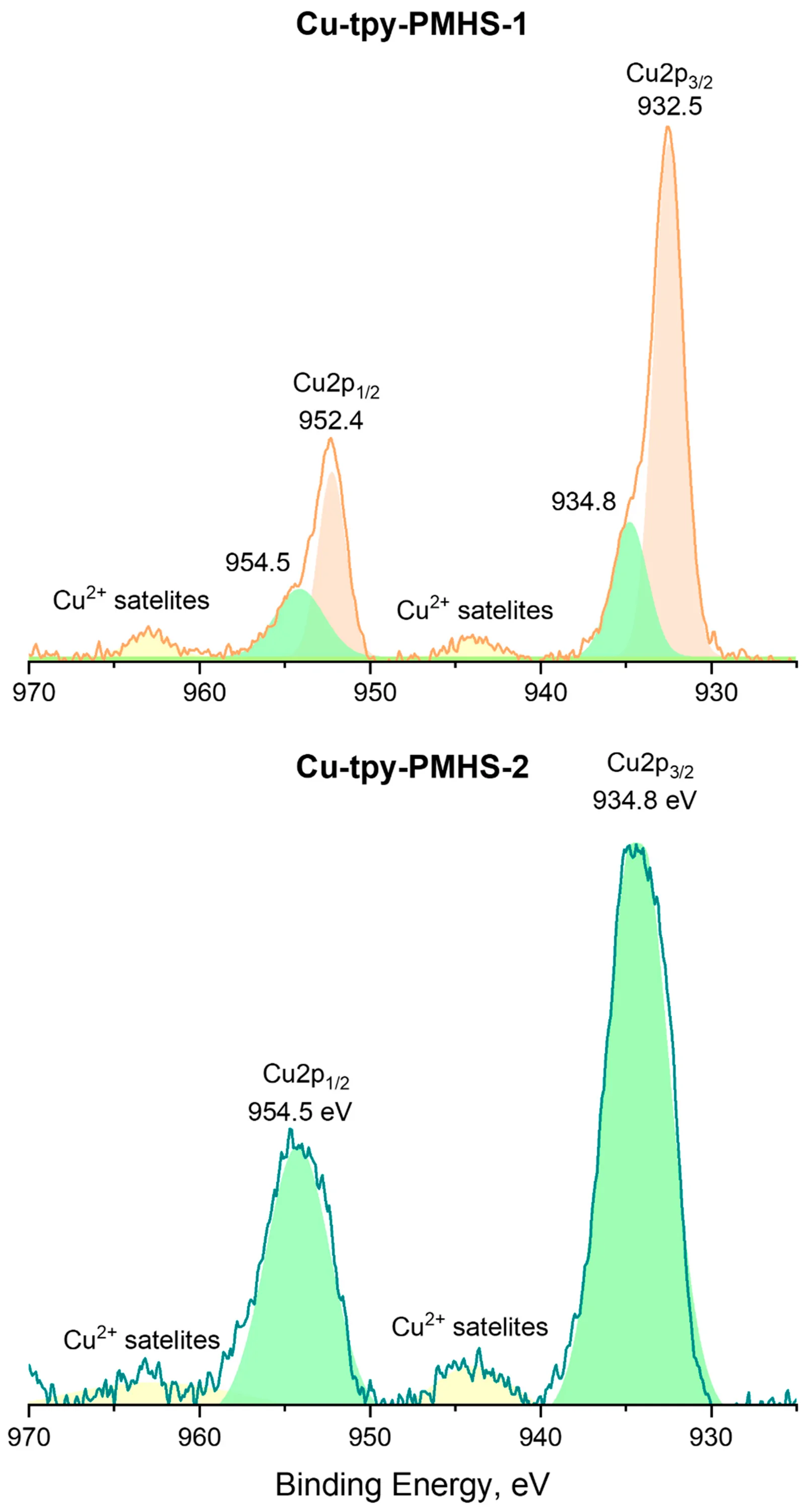
High-resolution Cu 2p XPS spectra of **Cu-tpy-PMHS-1** and **Cu-tpy-PMHS-2** samples.

The weak irreversible reduction at around −1.80 V can be attributed to a ligand-centered process of the terpyridine moiety [Cu^II^(tpy)]+e^−^⇌[Cu^II^(tpy^•−^)] or a further reduction involving copper [50,53]. Thus, the redox properties of **Cu-tpy-PMHS-2** are in good agreement with those reported for low molecular weight copper(II) complexes [50]. To correlate the electrochemical behavior with the oxidation states of copper centers present in PMCs, high-resolution XPS analysis of the Cu 2p region of **Cu-tpy-PMHS-1** and **Cu-tpy-PMHS-2** was additionally performed (Figure 9).

The Cu 2p spectra of **Cu-tpy-PMHS-1** and **Cu-tpy-PMHS-2** exhibited characteristic Cu 2p_3/2_ and Cu 2p_1/2_ signals located at approximately 932–935 and 952–955 eV, respectively. For **Cu-tpy-PMHS-1**, the Cu 2p_3/2_ peak could be described by two components centered at 932.5 and 934.8 eV, which can be assigned to Cu(I) and Cu(II) centers [54]. In addition to the main Cu 2p peaks, weak satellite features were detected at higher binding energies for both samples. Since **Cu-tpy-PMHS-2** was synthesized using the copper(II) precursor, the observed satellite contributions are consistent with the presence of Cu(II) atoms [54,55]. The appearance of similar satellite features in the spectrum of **Cu-tpy-PMHS-1**, which was prepared from the copper(I) salt, suggests partial oxidation of Cu(I) to Cu(II) on the PMC surface during preparation or subsequent exposure to air.

**Figure 10:**
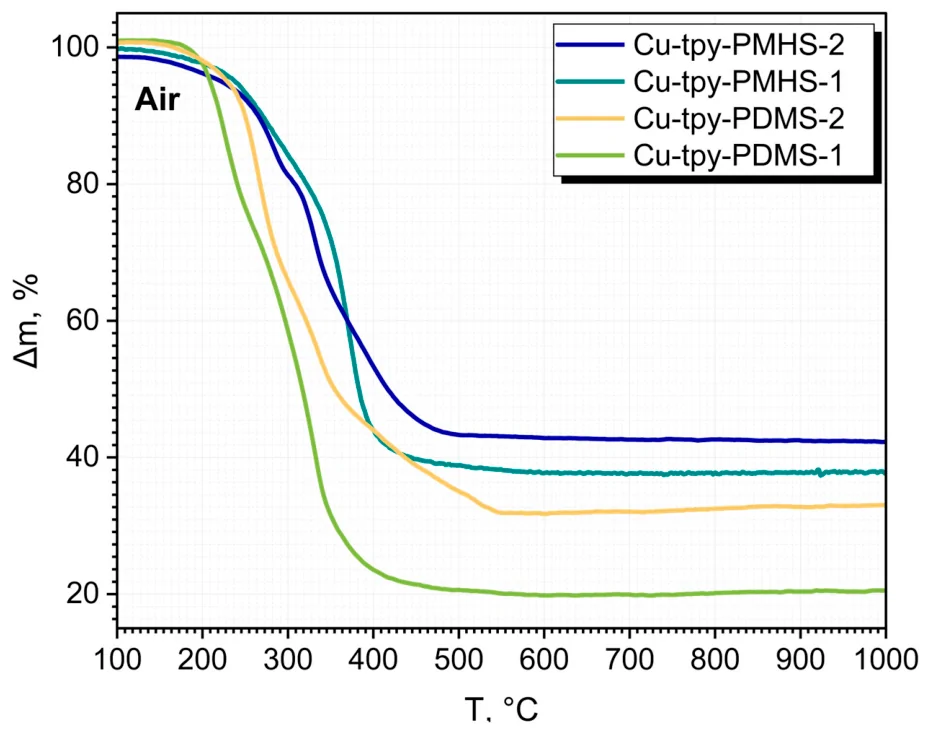
Thermal stability of copper-containing PMCs under air.

The thermal stability of all investigated polysiloxanes was evaluated by TGA under air over the temperature range of 100–1000 °C (Figure 10). TGA was carried out to determine the thermal stability and operational temperature range of the synthesized PMCs, which is relevant considering their potential use as catalysts. For **Cu-tpy-PDMS-1** and **Cu-tpy-PDMS-2** complexes, the 5% mass loss temperature (T_5%_) was found to be 209 °C and 229 °C, with final residues of 21% and 33%, respectively. The decomposition temperatures of the cross-linked **Cu-tpy-PMHS-1** and **Cu-tpy-PMHS-2** were quite similar, at 238 °C and 222 °C in air. The final residues of these samples were relatively higher (38–42%) at 1000 °C. In comparison, PMHS has the T_5%_ value of 159 °C, with the final residue of 11.1% [56]. Thus, the T_5%_ values of the synthesized copper-containing PMCs were 50–79 °C higher than that of the initial PMHS.

The residual masses of the obtained PMCs are in good agreement with previously reported values. In particular, copper coordination with a pyridylamine-functionalized hyperbranched polysiloxane resulted in an increased residual mass of up to 57%, attributed to the formation of strong metal–ligand bonds within the PMC framework [21]. Moreover, copper(II) complexes based on various siloxane-derived carboxylate ligands were shown to yield final residues of 22–65% at 450 °C in air [57].

Thus, copper-containing PMCs based on terpyridine-functionalized polysiloxanes were successfully synthesized and characterized, demonstrating the influence of metal coordination on polysiloxane functional properties.

### 3.2. Catalytic Activity of Copper-Containing Polysiloxanes

Copper-containing terpyridine complexes have demonstrated catalytic activity in numerous transformations, including radical trifluoromethylation [41], C–C cross-coupling reactions [58], aerobic oxidation of alcohols [4], and CuAAC [5]. While CuAAC is routinely applied to both small molecules and polymers under conventional solution conditions, increasing attention has recently been devoted to mechanochemical approaches due to their solvent-free nature and operational simplicity [46,59]. In the present study, polysiloxanes were successfully modified under mechanochemical conditions using [Cu(CH_3_CN)_4_]BF_4_ as a copper precursor (see Section 3.1.2. CuAAC Synthetic Approach). Furthermore, the catalytic performance of the resulting PMCs prepared from this copper source was evaluated.

**Figure 11:**
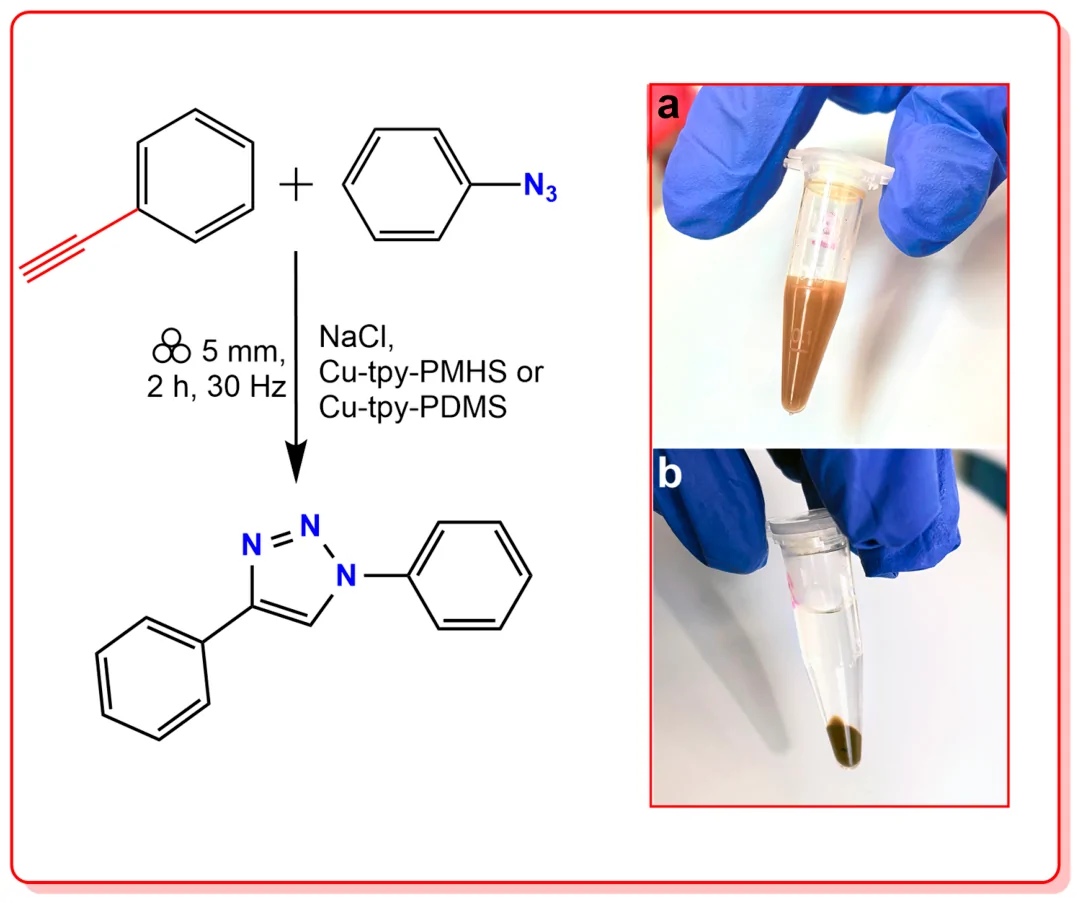
Model reaction between phenyl azide and phenylacetylene: (**a**) reaction mixture before centrifugation and (**b**) reaction mixture after centrifugation.

A model cycloaddition between phenyl azide and phenylacetylene was initially carried out in the presence of **Cu-tpy-PDMS-1** and **Cu-tpy-PMHS-1** (Figure 11). The selection of copper(I)-containing PMCs was motivated by the well-established catalytic activity of copper(I) derivatives in CuAAC reactions involving organic substrates [60]. Reagents were milled with NaCl and catalyst at 30 Hz for 2 h. First, the reaction was performed using the cross-linked **Cu-tpy-PMHS-1** sample, which exhibits poor solubility in organic solvents at r.t., enabling facile separation of the reaction product (Figure 11b). After completion of the reaction, the mixture was washed with deuterated CDCl_3_ and centrifuged, resulting in quantitative precipitation of the catalyst with NaCl. The mass of the recovered solid mixture remained nearly unchanged compared with the initial catalyst–NaCl mixture, confirming efficient separation. The formation of the target product was confirmed by ^1^H NMR spectroscopy (Figures S8 and S9), with an estimated yield of 20–25%.

For the **Cu-tpy-PDMS-1** sample, the procedure was the same: after completion of the reaction, the mixture was directly washed with CDCl_3_, centrifuged, and subsequently analyzed by NMR spectroscopy (Figures S8 and S9). The reaction yield, calculated from the integral intensities of the phenylacetylene and triazole proton resonances, reached 80%.

The catalytic reactions using **Cu-tpy-PMHS-2** and **Cu-tpy-PDMS-2** were additionally investigated under the same conditions. The formation of the target product was confirmed by ^1^H NMR spectroscopy (Figure S10). The average product yields obtained with **Cu-tpy-PDMS-2** and **Cu-tpy-PMHS-2** as catalysts were 70% and 24%, respectively. These results demonstrate that, under the applied reaction conditions, the oxidation state of copper does not result in a significant reduction in catalytic activity. The average yields of all catalytic experiments are summarized in Table S1.

The higher yields obtained with **Cu-tpy-PDMS-1** and **Cu-tpy-PDMS-2** may be associated with differences in polymer architecture. The cross-linked structure of **Cu-tpy-PMHS-1** or **Cu-tpy-PMHS-2** could restrict substrate diffusion and reduce the accessibility of copper coordination sites, which may account for the lower catalytic efficiency compared with **Cu-tpy-PDMSs**. A control experiment was also performed to verify whether the reaction could proceed in the absence of the catalyst (**Cu-tpy-PDMS-1** or other catalysts). No cycloaddition product was detected under identical reaction conditions, confirming the essential role of the copper-containing PMCs in this transformation.

To evaluate the stability of the copper-containing PMCs during mechanochemical reaction, the recovered solids (the mixture of the catalyst and NaCl) were additionally analyzed by XPS. The XPS spectra (Figure S11) of the recovered **Cu-tpy-PDMS-1** and **Cu-tpy-PMHS-1** samples exhibited signals corresponding to those of the initial copper-containing PMCs. The Cu 2p signals were detected at 932.49–932.63 eV, while signals attributed to NaCl were also observed. Also, comparison of the copper atomic concentrations before and after the catalytic reaction revealed no significant loss of metal. For **Cu-tpy-PMHS-1**, the copper atomic concentration decreased slightly from 1.4% to 1.2%. For **Cu-tpy-PDMS-1**, the copper atomic concentration after the reaction was 2.1%, which is comparable to that of the initial complex.

Several examples of copper-containing PMCs based on polysiloxanes have been reported as catalysts for click reactions. For example, copper-containing hyperbranched polysiloxane was used as a heterogeneous catalyst for the synthesis of 5-substituted 1H-tetrazoles [21]. In another study, diamine-functionalized polysiloxane was employed as a ligand for CuAAC, leading to the formation of the desired products in excellent yields [61]. However, all known polysiloxane-based copper catalysts have involved reactions carried out in solution. In contrast, the present study demonstrates, for the first time, the successful application of polysiloxane-based PMC as a catalyst for mechanochemical azide–alkyne cycloaddition, highlighting its potential as an efficient heterogeneous catalyst.

## 4. Conclusions

For the first time, copper-containing PMCs based on terpyridine-functionalized polysiloxanes via hydrosilylation were successfully synthesized. A comparative investigation of synthetic approaches identified hydrosilylation as the most suitable method for incorporating terpyridine fragments into polysiloxane chains, enabling access to polymeric ligands with different architectures (**tpy-PDMS** and **tpy-PMHS**). Subsequent coordination with copper(I) and copper(II) precursors provided four structurally diverse PMCs (**Cu-tpy-PDMS-1**, **Cu-tpy-PDMS-2**, **Cu-tpy-PMHS-1**, and **Cu-tpy-PMHS-2**).

The incorporation of copper was confirmed by spectroscopic and electrochemical techniques. The obtained PMCs contained 5.3–10.5 wt.% of copper according to EDX analysis, while XPS measurements confirmed the presence of surface copper centers. The residual Si–H groups in **tpy-PMHS** and the corresponding **Cu-tpy-PMHS** complexes enabled self-cross-linking upon heating, producing cross-linked networks. The cross-linked **Cu-tpy-PMHS-2** sample exhibited redox activity, demonstrating the potential of polysiloxane-based PMCs as electroactive materials. In addition, all synthesized PMCs exhibited good thermal stability, with decomposition temperatures (T_5%_) ranging from 209 to 238 °C and residual masses of 21–42% at 1000 °C in air.

The catalytic potential of the copper-containing PMCs was demonstrated in the mechanochemical CuAAC model reaction between phenyl azide and phenylacetylene. The non-cross-linked **Cu-tpy-PDMS-1** and **Cu-tpy-PDMS-2** exhibited the highest catalytic activity, providing 80% and 70% yields, respectively, after 2 h. The developed PMCs offer several advantages as heterogeneous catalysts, notably their easy separation from the reaction mixture and good thermal stability. Furthermore, the redox activity of copper-containing PMCs suggests potential applications beyond the catalytic utilization investigated in this study. Although the yield did not exceed 80%, the proposed mechanochemical approach offers an important advantage by eliminating the use of solvent during the reaction. Thus, these findings represent the first example of a polysiloxane-based polymer–metal complex employed as a catalyst for a mechanochemical transformation, opening new opportunities for developing heterogeneous polymer catalysts and redox-active functional materials.

## Data Availability

The original contributions presented in this study are included in the article/Supplementary Material. Further inquiries can be directed to the corresponding author.

## Supplementary Materials

The following supporting information can be downloaded at: https://www.mdpi.com/article/10.3390/polym18202506/s1.
