# Supplementary material for: Some Synthetic Approaches for Copper-Containing Terpyridine-Functionalized Polysiloxane Complexes
Source: Polymers (Basel). 2026 Oct 13;18(20):2506. doi: 10.3390/polym18202506 (PMC13708818; doi:10.3390/polym18202506)
Supplement: Supplementary file 1 [file polymers-18-02506-s001.zip › polymers-4580467-supplementary.pdf]

Supporting Information

Some Synthetic Approaches for Copper-Containing Terpyridine-Functionalized Polysiloxane Complexes

*Sofia S. Kryukova, Elena R. Votchel, Sergey V. Baykov, Vadim P. Boyarskiy, and Regina M. Islamova**

Institute of Chemistry, Saint Petersburg State University, 7/9 Universitetskaya Emb., Saint Petersburg, Russia, 199034

*Corresponding author

E-mail address: r.islamova@spbu.ru (R.M.I.).

Contents

S1 Characterization of Synthesised Compounds 2

S2 Catalytic experiments 7

S3 Synthesis via CuAAC Reaction 10

S3.1. CuAAC Reaction Catalyzed by $[Cu(CH_3CN)_4]BF_4$ 10

S3.2. CuAAC Reaction Catalyzed by CuBr 11

S3.3. CuAAC Reaction between N_3-PDMS and Phenylacetylene 12

S3.4. Mechanochemical CuAAC Reaction 13

S3.5. Model Reaction between Terpyridine Ligand and $[Cu(CH_3CN)_4]BF_4$ 14

S1 Characterization of Synthesised Compounds

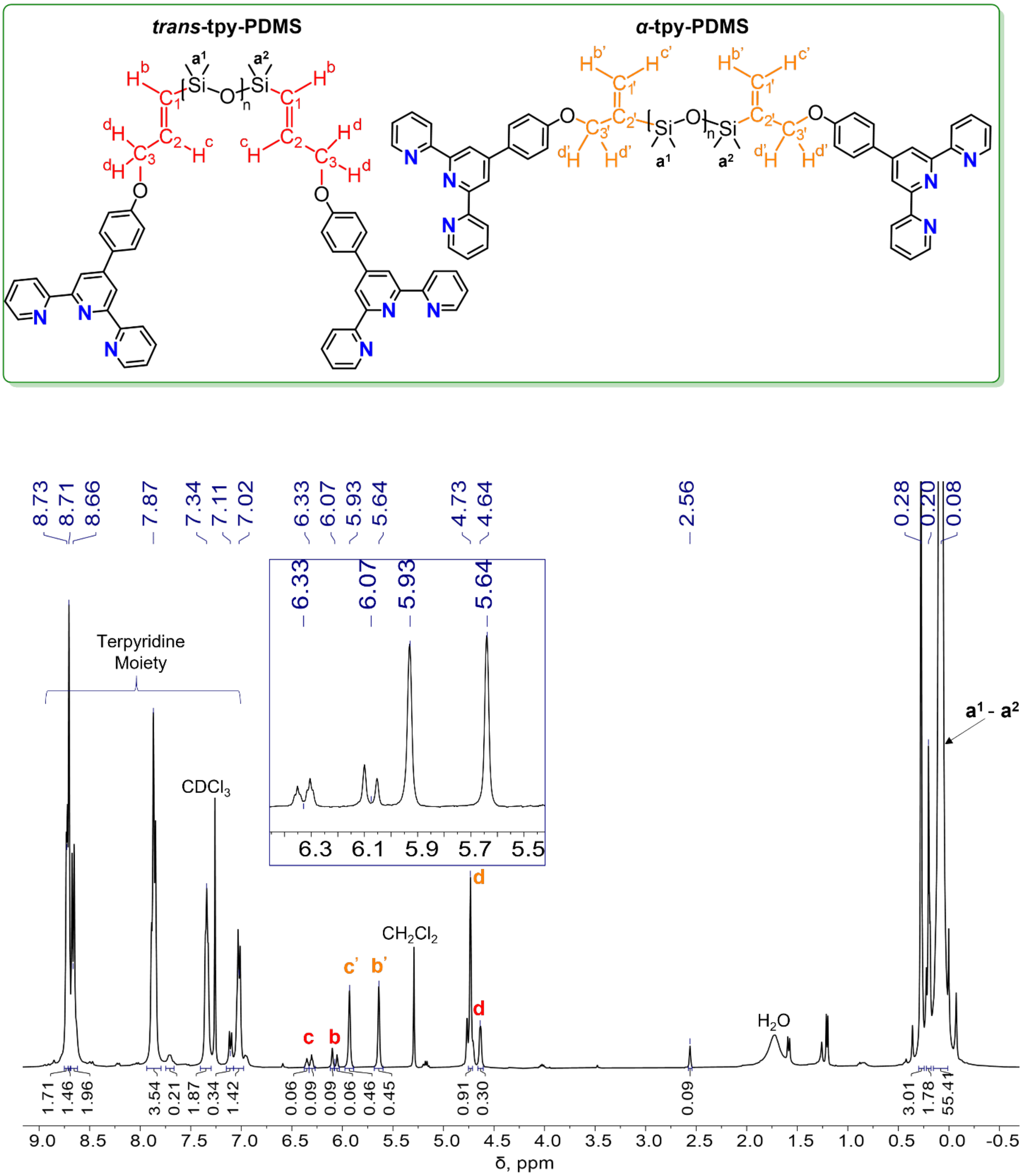

Figure S1. Assignment of the liquid-state ^{1}H NMR spectrum of **tpy-PDMS** (*α*- and *trans*-isomers) recorded in $CDCl_3$.

a

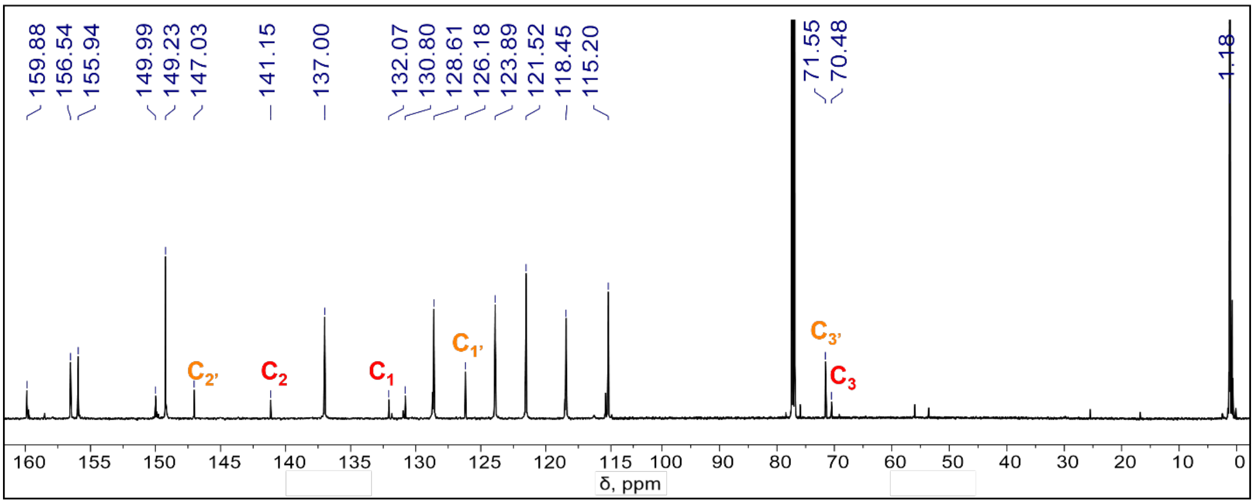

b

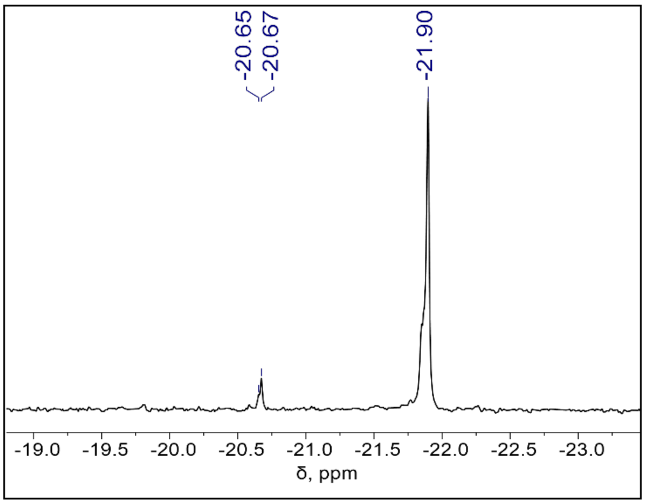

Figure S2. Liquid-state $^{13}C\{^1H\}$ (**a**) and $^{29}Si\{^1H\}$ (**b**) NMR spectra of **tpy-PDMS** recorded in $CDCl_3$.

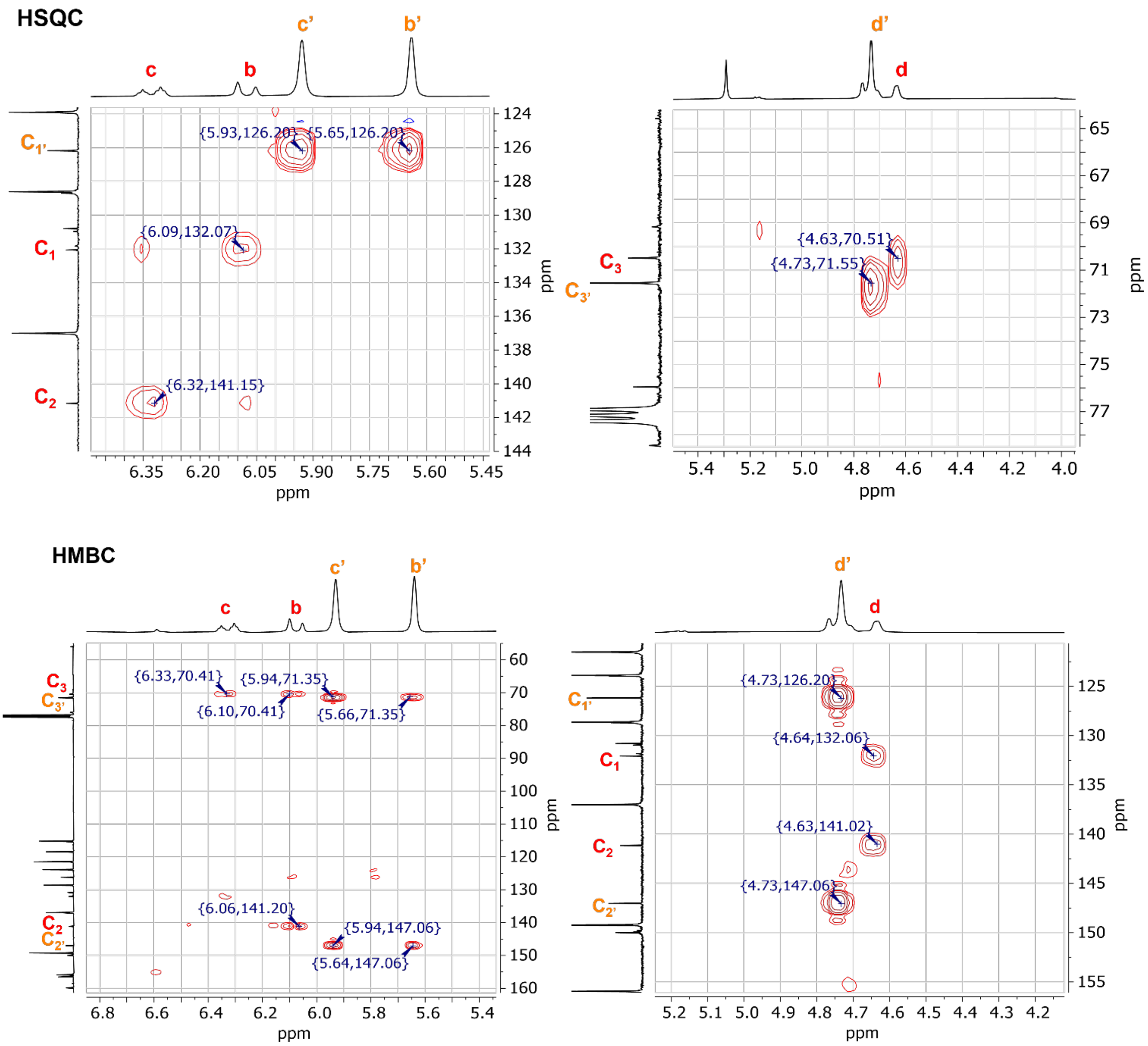

Figure S3. Assignment of the liquid-state HSQC and HMBC NMR spectra of **tpy-PDMS** (*α*- and *trans*-isomers) recorded in $CDCl_3$.

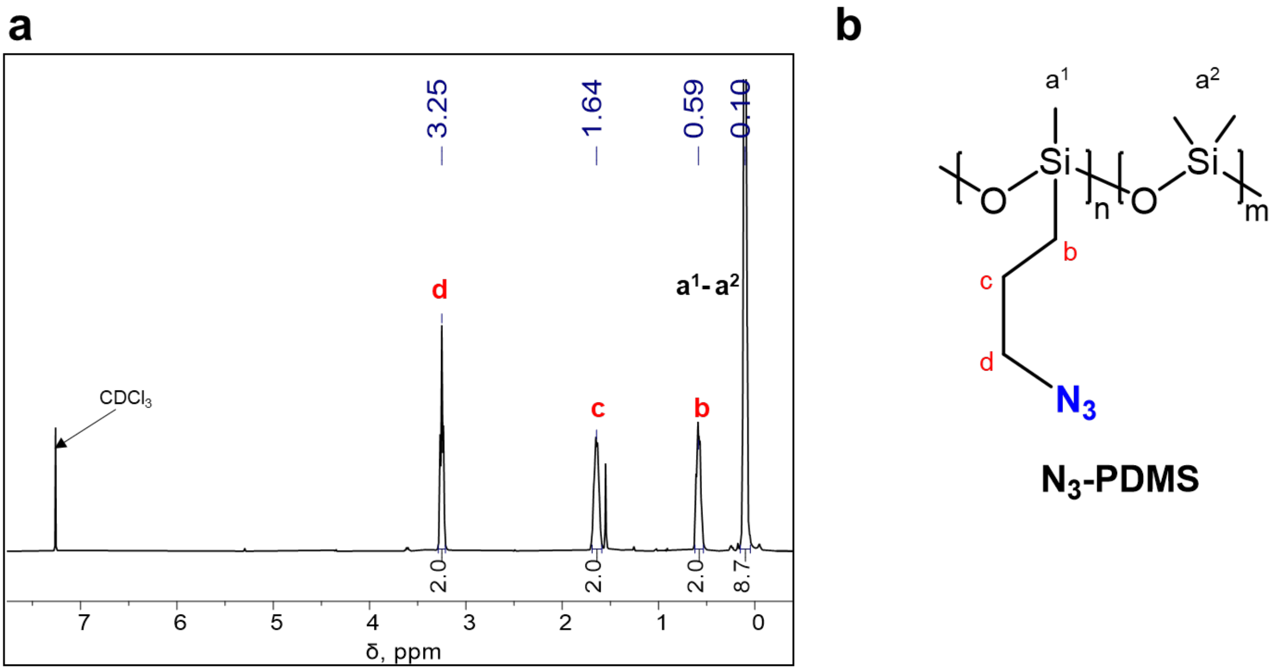

Figure S4. Liquid-state ^{1}H NMR spectra of **N$_3$-PDMS** recorded in $CDCl_3$.

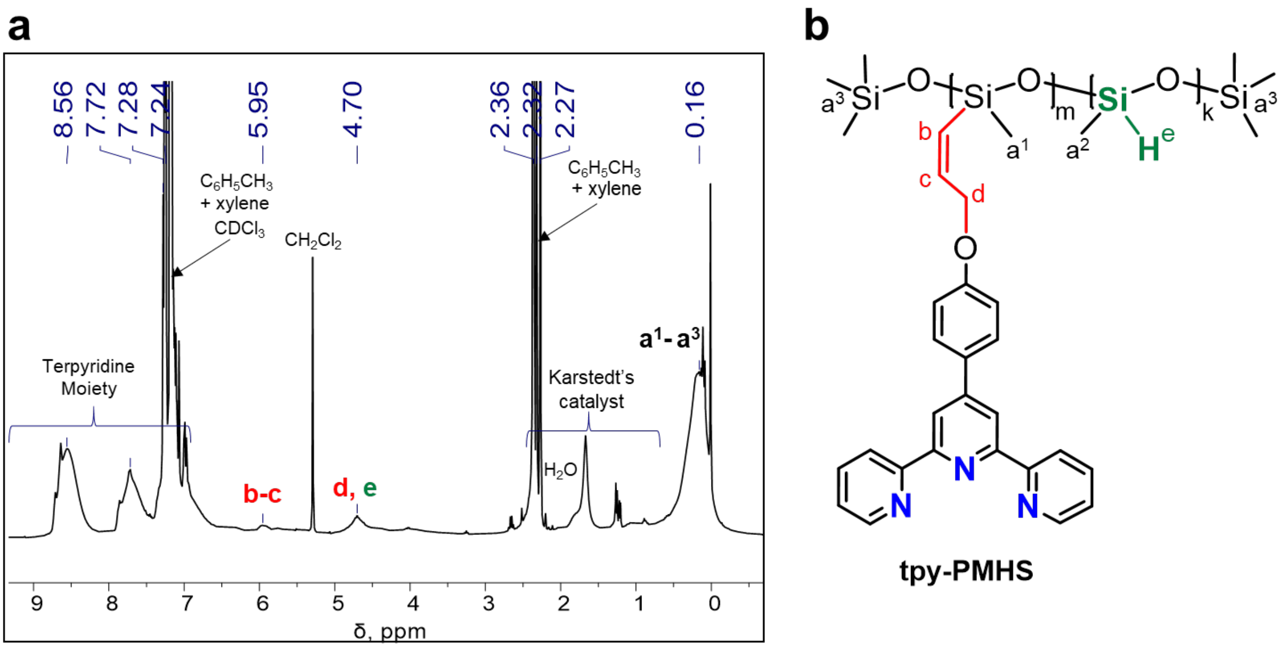

Figure S5. Liquid-state ^{1}H NMR spectra of **tpy-PMHS** solution registered in $CDCl_3$ (**a**) and proposed structure of **tpy-PMHS** (**b**).

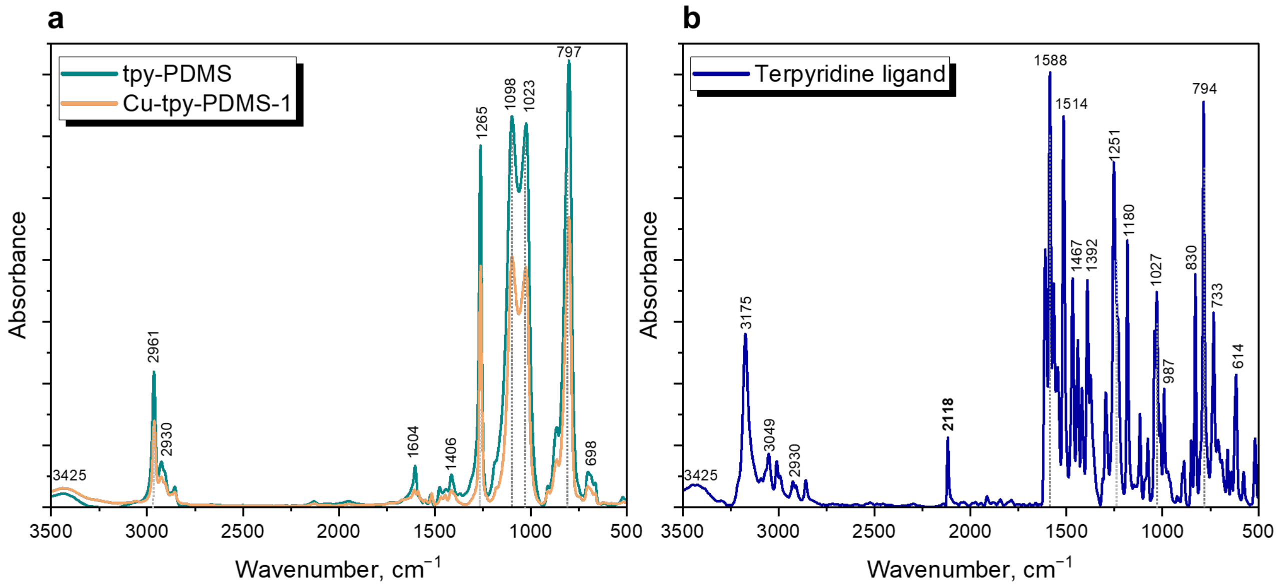

Figure S6. FTIR spectra of **tpy-PDMS**, **Cu-tpy-PDMS-1** (**a**), and terpyridine ligand (**b**).

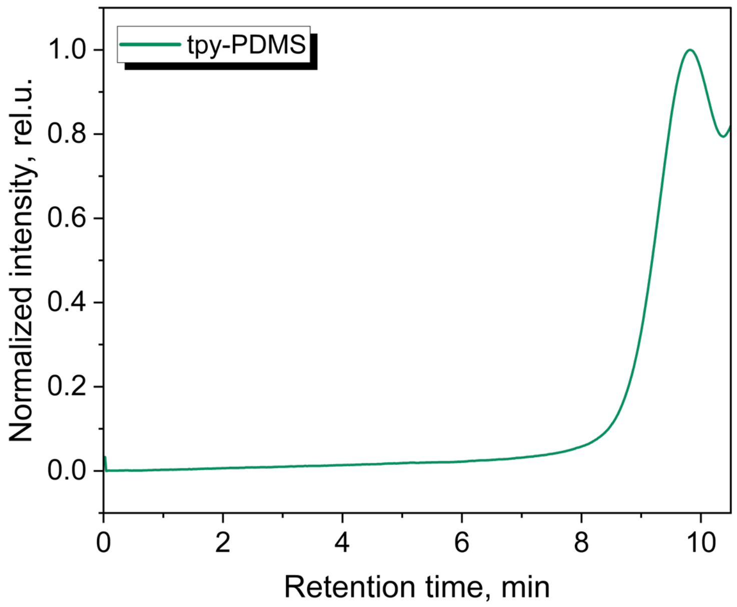

Figure S7. GPC curve of **tpy-PDMS**.

S2 Catalytic experiments

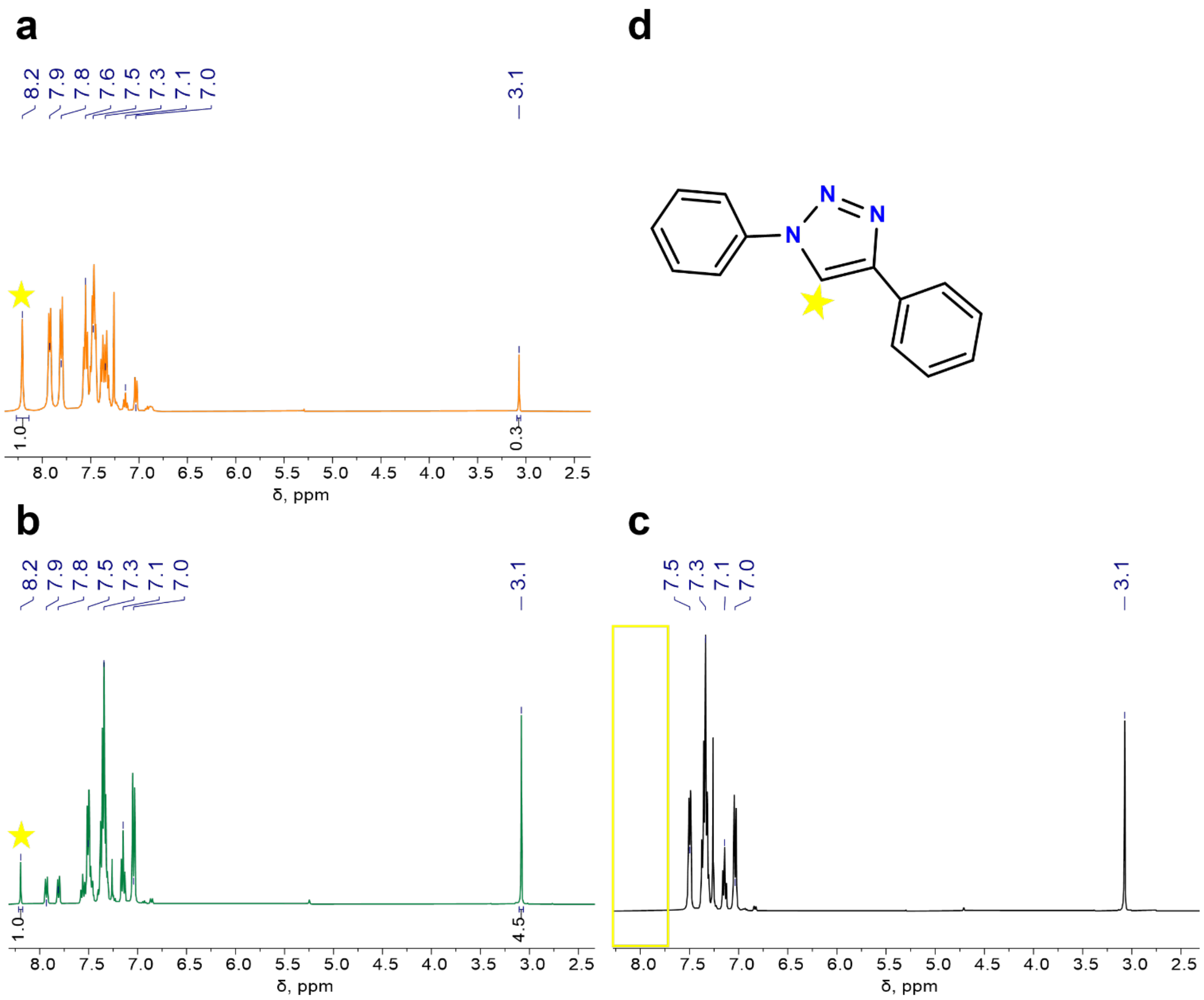

Figure S8. Liquid-state ^{1}H NMR spectra of the reaction mixtures of phenyl azide and phenylacetylene in the presence of different catalysts: **Cu-tpy-PDMS-1** (**a**), **Cu-tpy-PMHS-1** (**b**), as well as the uncatalyzed reaction (**c**); chemical structure of the obtained product (**d**).

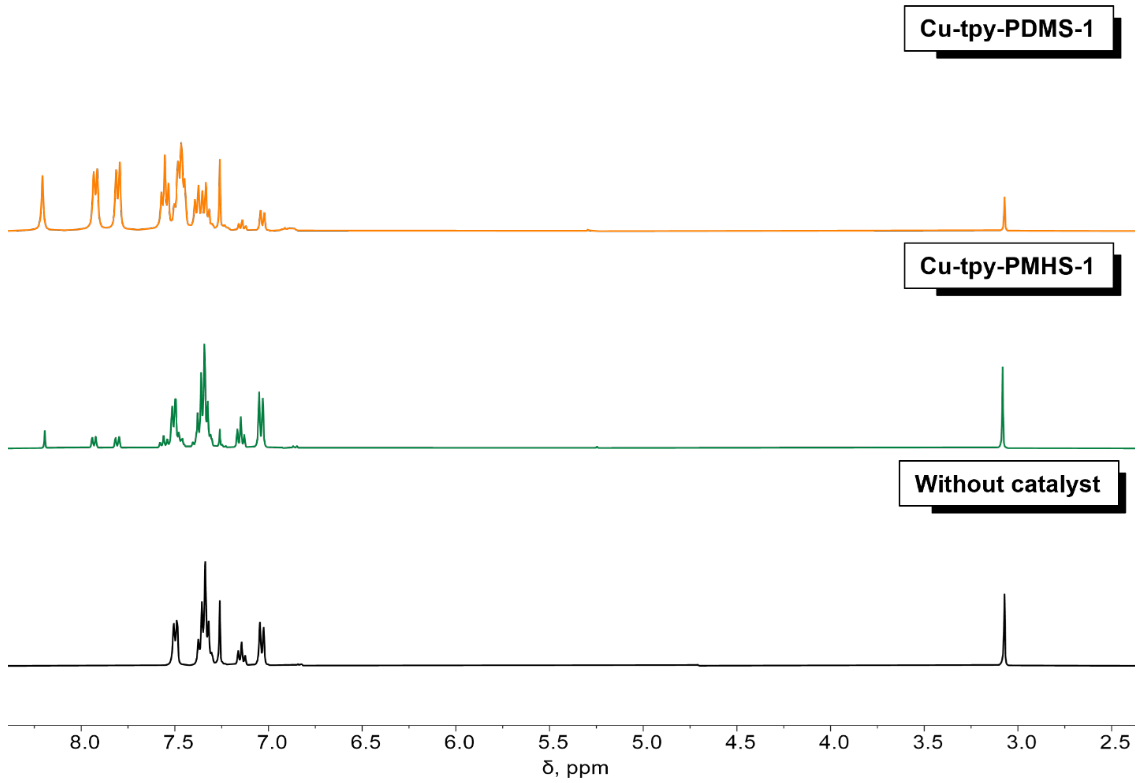

Figure S9. Comparison of ^{1}H NMR spectra of the reaction mixtures of phenyl azide and phenylacetylene obtained in the presence of **Cu-tpy-PDMS-1**, **Cu-tpy-PMHS-1**, and without catalyst.

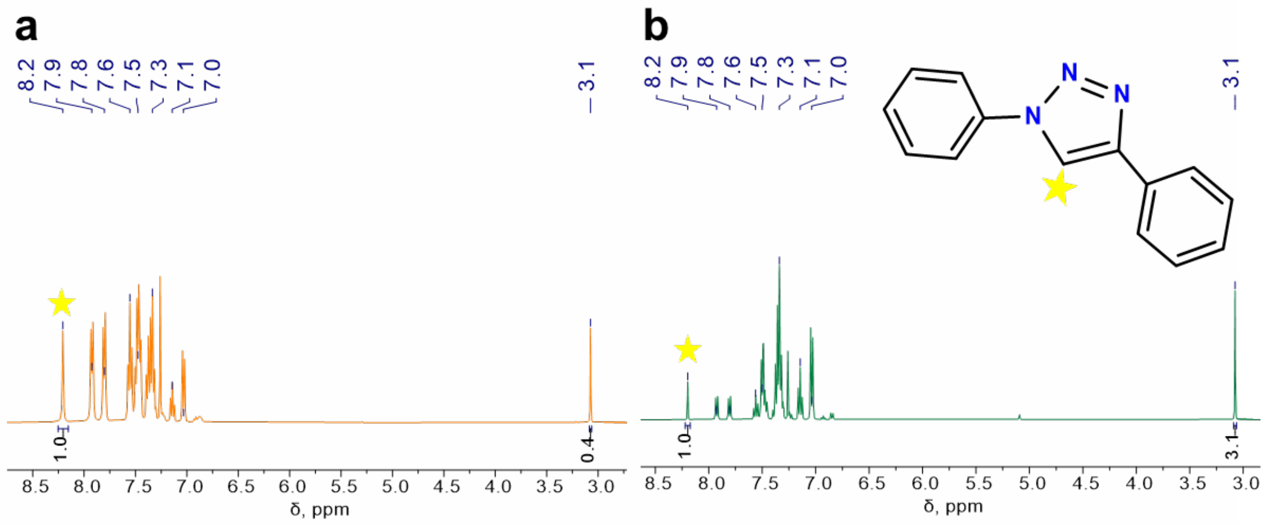

Figure S10. Liquid-state ^{1}H NMR spectra of the reaction mixtures of phenyl azide and phenylacetylene in the presence of different catalysts: **Cu-tpy-PDMS-2** (**a**) and **Cu-tpy-PMHS-2** (**b**).

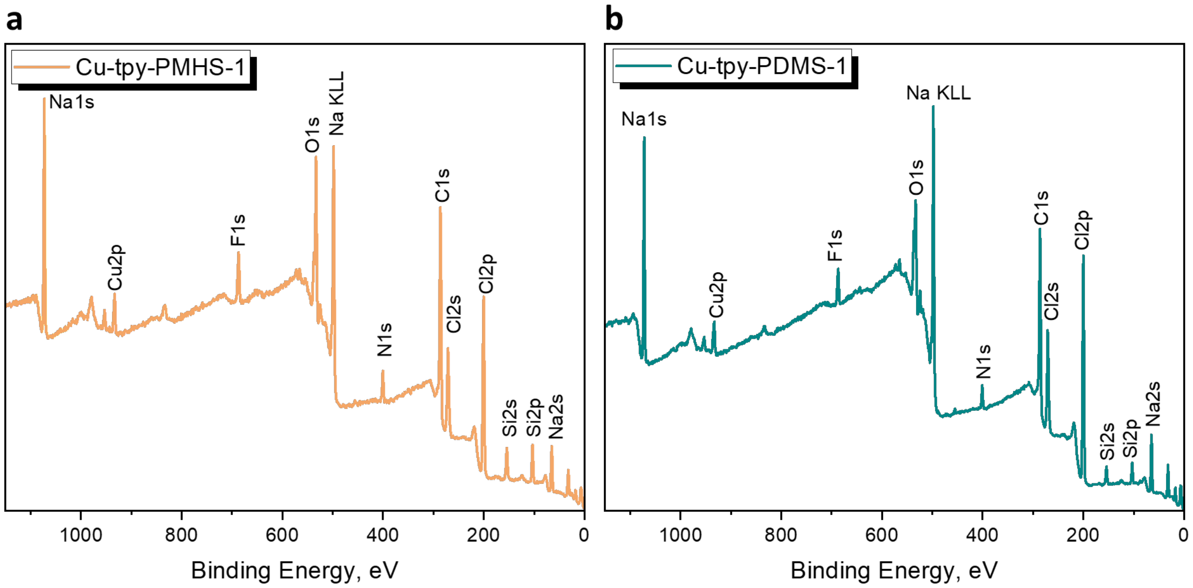

Figure S11. XPS survey spectra of recovered solids after catalytic mechanochemical experiments: **Cu-tpy-PMHS-1** (**a**) and **Cu-tpy-PDMS-1** (**b**) catalysts.

Table S1. Average yields with error bars for three repeated catalytic reactions using different catalysts.

Catalyst	Yield, %
**Cu-tpy-PDMS-1**	80±1
**Cu-tpy-PDMS-2**	70±2
**Cu-tpy-PMHS-1**	21±4
**Cu-tpy-PMHS-2**	24±1

S3 Synthesis via CuAAC Reaction

S3.1. CuAAC Reaction Catalyzed by $[Cu(CH_3CN)_4]BF_4$

N_3-PDMS (88 mg, 0.38 mmol, corresponding to 50 mol.% azide-functionalized siloxane units) and 4'-(4-(prop-2-yn-1-yloxy)phenyl)-2,2':6',2''-terpyridine (276 mg, 0.76 mmol) were dissolved in $CHCl_3$ (17 mL) and transferred to a Schlenk tube. The solution was purged with argon for 15 min, followed by dropwise addition of a degassed CH_3CN solution of $[Cu(CH_3CN)_4]BF_4$ (238 mg, 0.76 mmol in 4 mL) under an inert atmosphere. Immediate precipitation occurred upon mixing, accompanied by a color change from brown to green. The reaction mixture was heated at 70 °C for four days under an inert atmosphere. The precipitate and supernatant were subsequently separated and analyzed independently. The green precipitate was isolated by centrifugation (2500 rpm, 5 min) and dried under vacuum, while the supernatant was concentrated under reduced pressure (5 mbar) at 50 °C to afford a viscous brown substance.

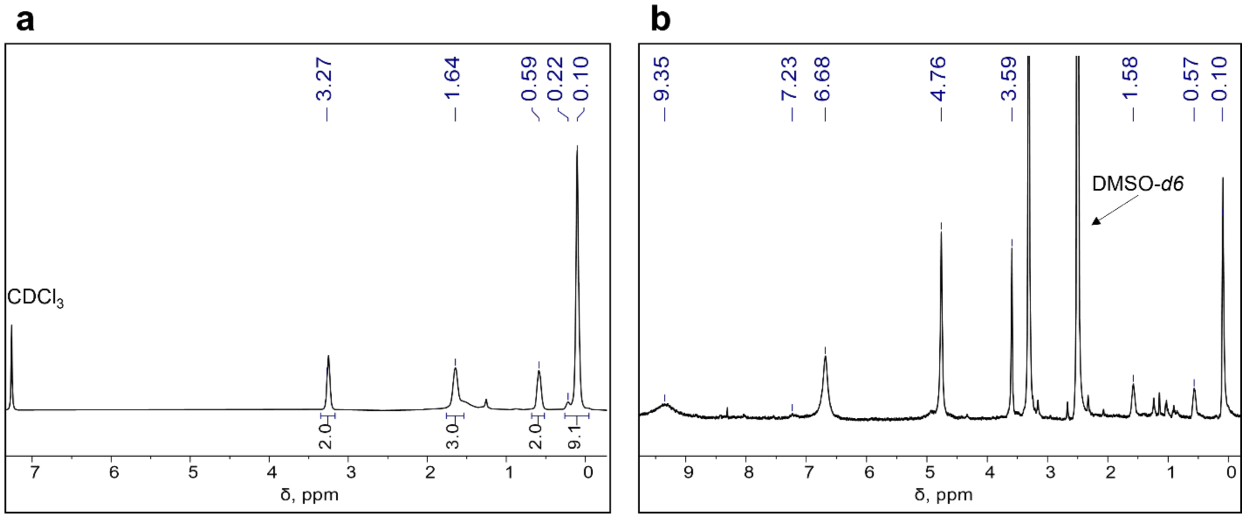

Figure S12. Liquid-state ^{1}H NMR spectra of the separated supernatant (**a**, recorded in $CDCl_3$) and the precipitate (**b**, recorded in DMSO-*d6*).

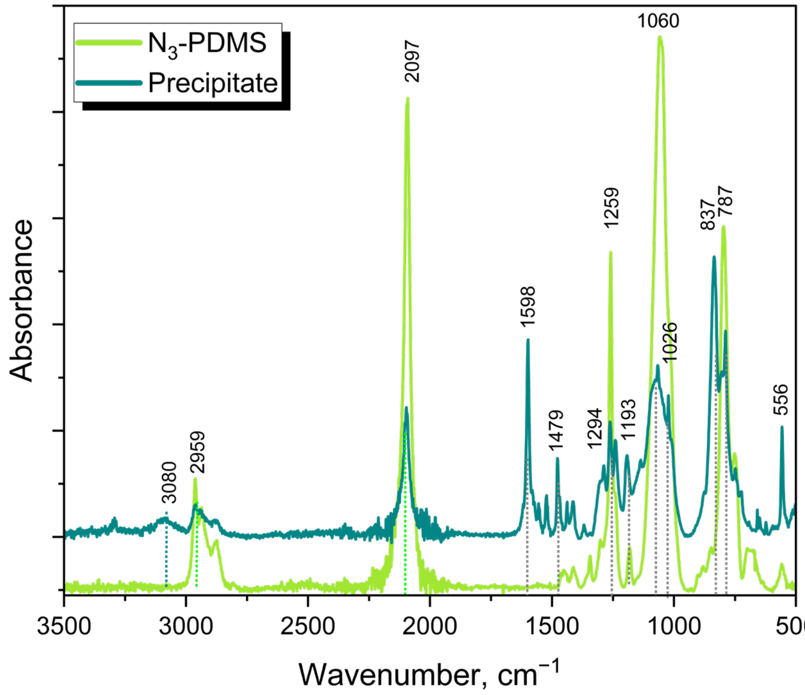

Figure S13. FTIR spectra of **N_3-PDMS** and the precipitated product.

S3.2. CuAAC Reaction Catalyzed by CuBr

The reaction was performed under conditions similar to those described in Section S3.1, with minor modifications. **N_3-PDMS** (103 mg, 0.45 mmol of azide groups; 50 mol.% azide-substituted siloxane units) and 4'-(4-(prop-2-yn-1-yloxy)phenyl)-2,2':6',2"-terpyridine (321.9 mg, 0.89 mmol) were dissolved in $CHCl_3$ (17 mL). CuBr (127.1 mg, 0.89 mmol) was dissolved in DMF (4 mL) and added to the reaction mixture together with TMEDA (0.1 mL) in a Schlenk tube under an inert atmosphere. The reaction mixture was heated at 70 °C for four days. Precipitation of a green solid was observed upon mixing. After the reaction was complete, the mixture was centrifuged, and the solvents were removed under reduced pressure (5 mbar) at 50 °C. The precipitated product was dried on a rotary evaporator at 50 °C for 2 h.

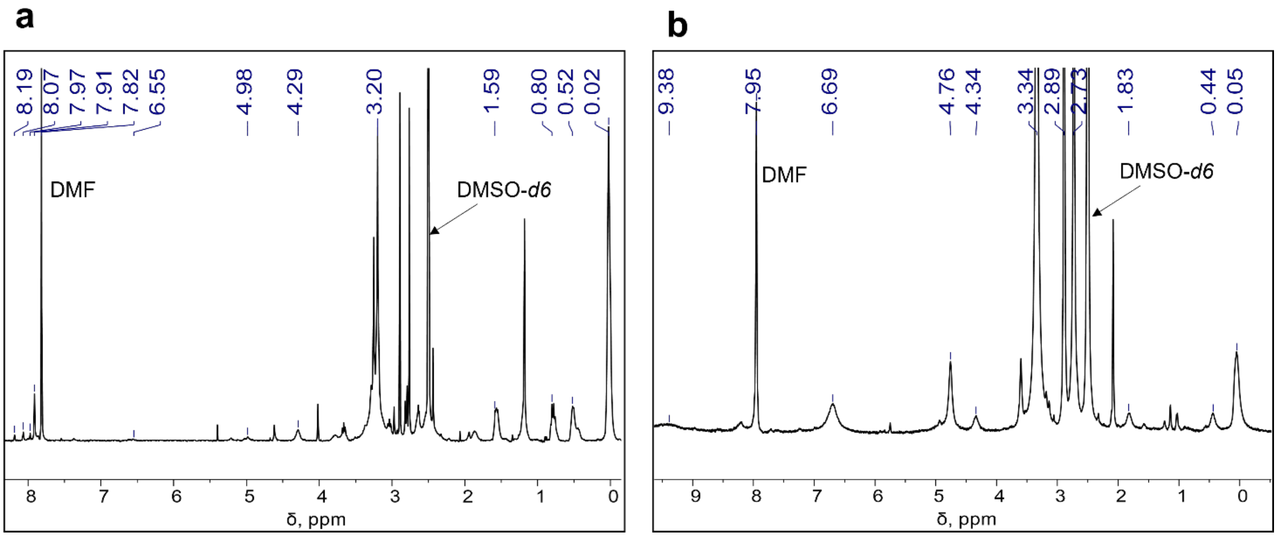

Figure S14. Liquid-state ^{1}H NMR spectra of the separated supernatant (**a**, recorded in a mixture of DMSO–*d6*/$CDCl_3$) and precipitate (**b**, recorded in DMSO-*d6*).

S3.3. CuAAC Reaction between N_3-PDMS and Phenylacetylene

Mechanochemical CuAAC reactions between **N_3-PDMS** and phenylacetylene were carried out using two different copper catalysts: $Cu(NO_3)_2 \cdot 3H_2O$ and $[Cu(CH_3CN)_4]BF_4$. **N_3-PDMS** (0.14 mmol of azide groups; 31.6 mg for the reaction catalyzed by $[Cu(CH_3CN)_4]BF_4$ and 30.2 mg for the reaction catalyzed by $Cu(NO_3)_2 \cdot 3H_2O$) and phenylacetylene (0.14 mmol; 14.6 mg and 14.0 mg, respectively) were loaded into stainless-steel vessels (1.5 mL). The copper catalyst (0.5 equiv. relative to **N_3-PDMS**), either $Cu(NO_3)_2 \cdot 3H_2O$ (0.07 mmol, 17.4 mg) or $[Cu(CH_3CN)_4]BF_4$ (0.07 mmol, 22.6 mg), together with NaCl (50 mg) as a filler, was added to the vessels. The reactions were performed in a vibrational ball mill at 30 Hz for 1 h. After milling, the reaction mixtures were extracted with CH_2Cl_2 and subsequently washed with an aqueous EDTA solution and water to remove residual copper salts. The organic layers were dried over anhydrous Na_2SO_4 for 2 h, and the solvent was removed under reduced pressure (5 mbar) at 40 °C for 1 h. The products were obtained as viscous light-yellow liquids.

According to ^{1}H NMR spectroscopy (Figure S15), the substitution degree was 83% and 80% for the reactions catalyzed by $[Cu(CH_3CN)_4]BF_4$ and $Cu(NO_3)_2 \cdot 3H_2O$, respectively.

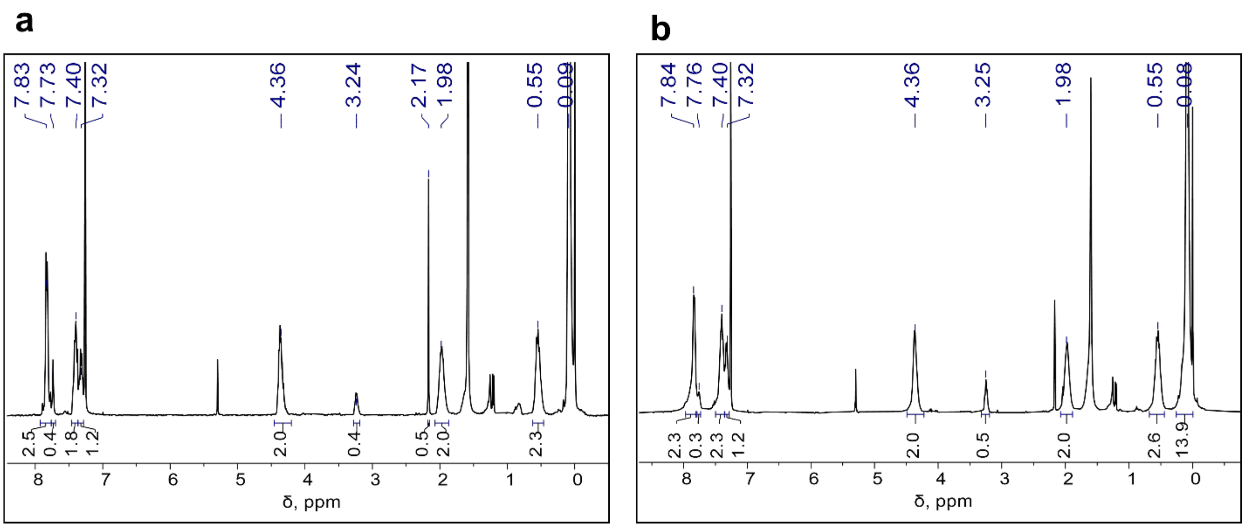

Figure S15. Liquid-state ^{1}H NMR spectra of modified **N_3-PDMS** using $[Cu(CH_3CN)_4]BF_4$ (**a**) and $Cu(NO_3)_2 \cdot 3H_2O$ (**b**) as catalysts, recorded in $CDCl_3$.

S3.4. Mechanochemical CuAAC Reaction

4'-(4-(prop-2-yn-1-yloxy)phenyl)-2,2':6',2''-terpyridine (50 mg, 0.14 mmol), **N_3-PDMS** (32 mg, 0.14 mmol of azide groups; 50 mol.% azide-substituted siloxane units), and $[Cu(CH_3CN)_4]BF_4$ (43 mg, 0.14 mmol) were placed in a stainless-steel vessel (1.5 mL). A stainless-steel ball (5 mm) was then added to the jar, which was subsequently sealed under air. The reaction vessel was placed in a vibrational ball mill (Figure S16, b) for 2 h at a frequency of 30 Hz. After completion of the reaction, an appropriate amount of the sample was dissolved in DMSO-*d6* and analyzed by NMR spectroscopy (Figure S16, a).

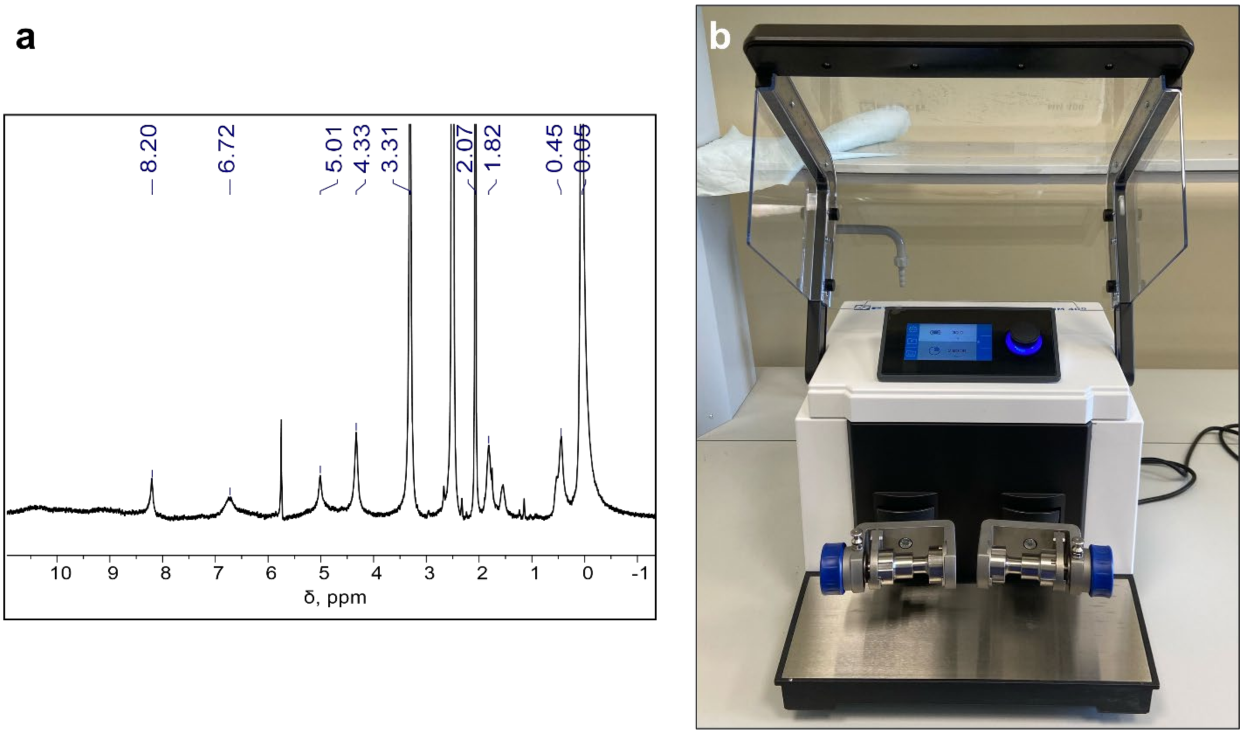

Figure S16. Liquid-state ^{1}H NMR spectra of the reaction mixture in DMSO–*d6* (**a**) and photograph of the vibrational ball mill (**b**).

S3.5. Model Reaction between Terpyridine Ligand and $[Cu(CH_3CN)_4]BF_4$

A solution of $[Cu(CH_3CN)_4]BF_4$ (10 mg, 0.03 mmol) in DMSO-*d6* (300 µL) was purged with argon for 10 min and subsequently added to a solution of 4'-(4-(prop-2-yn-1-yloxy)phenyl)-2,2':6',2''-terpyridine (11.6 mg, 0.03 mmol) in DMSO–*d6* under an argon atmosphere. Upon mixing, an immediate color change of the solution to brown was observed, consistent with the formation of the copper(I)–terpyridine complex. The ^{1}H NMR spectra were recorded at 20 and 60 °C (Figure S17).

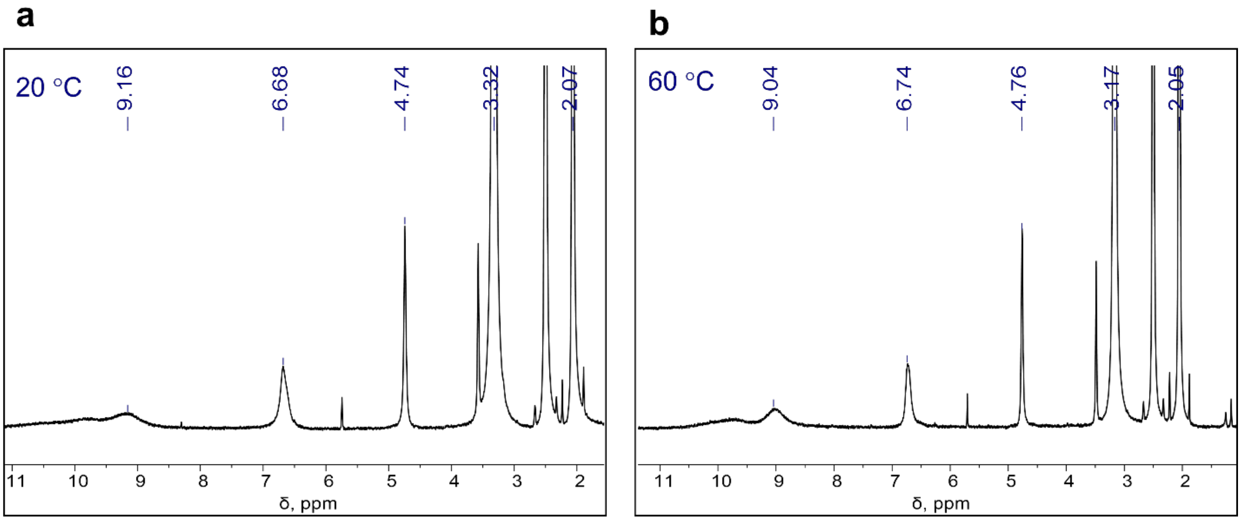

Figure S17. Liquid-state ^{1}H NMR spectra of the reaction mixture recorded in DMSO-*d6* at 20 °C (**a**) and 60 °C (**b**).
